# Morphogenesis of moss leaf-like organs through variations in deeply shared developmental principles

**DOI:** 10.1126/sciadv.aee6959

**Published:** 2026-04-15

**Authors:** Wenye Lin, Loann Collet, Laure Mancini, Mandar Deshpande, Brendan Lane, Benjamin P. Lapointe, Agnieszka Bagniewska-Zadworna, Anne-Lise Routier-Kierzkowska, Richard S. Smith, Yoan Coudert, Daniel Kierzkowski

**Affiliations:** ^1^ Institut de Recherche en Biologie Végétale, Département de Sciences Biologiques, Université de Montréal, 4101 Sherbrooke St E, Montréal, QC, H1X 2B2 Canada.; ^2^ Laboratoire Reproduction et Développement des Plantes, ENS de Lyon, CNRS, Université Claude Bernard Lyon 1, INRAE, Lyon 69007, France.; ^3^ Computational and Systems Biology, John Innes Centre, Norwich Research Park, Norwich NR4 7UH, UK.; ^4^ Department of General Botany, Institute of Experimental Biology, Faculty of Biology, Adam Mickiewicz University, Poznań, Poland.

## Abstract

Leaves and leaf-like organs with laminar structures and determinate growth arose multiple times independently in land plants. The cellular basis of leaf development is well characterized in flowering plants, and molecular studies have shown that the plant hormone auxin plays a central role in this process, orchestrating cellular growth and differentiation. Auxin is also crucial for the formation of phyllids, the leaf-like organs of bryophytes, yet its precise role in morphogenesis remains unclear. More broadly, whether similar developmental principles are shared across distantly related evolutionary lineages is unknown. Here, we combine live-imaging, genetics, pharmacological treatments, and modeling to investigate the cellular and molecular basis of phyllid development in the model moss *Physcomitrium patens*. By tracking phyllid morphogenesis from a single initial cell to full maturity, we uncover the cellular growth dynamics underlying organ development. We demonstrate that auxin spatially inhibits cell divisions and promotes cellular elongation and differentiation. However, unlike in vascular plants, moss PIN transporters do not participate in polar auxin transport during phyllid development but mainly reduce intracellular auxin concentration. These findings indicate that while auxin’s role in organogenesis is conserved, its transport mechanisms have diverged across land plants. Overall, our study reveals shared principles of planar organ morphogenesis, highlighting how the repeated deployment of similar developmental strategies, with lineage-specific variations, drove the convergent evolution of leaves and leaf-like organs.

## Teaser

Auxin modulates a conserved developmental program in moss phyllids by spatiotemporally controlling cell division and growth.

## INTRODUCTION

Leaves are specialized photosynthetic structures that evolved multiple times independently in the sporophyte generation of vascular plants (*1*, *2*). They range from large, structurally complex organs, referred to as “true” leaves in flowering plants, to smaller, anatomically simple structures, such as lycophyte microphylls. The repeated emergence of leaves and other leaf-like organs has involved the recruitment of both conserved and distinct genetic mechanisms (*3*, *4*).

Beyond vascular plants, leaf-like organs known as phyllids evolved convergently in the gametophyte generation of bryophytes, including leafy liverworts and mosses. Phyllids are small lateral appendages that develop at the apex of leafy shoots called gametophores. In the model moss species *Physcomitrium patens*, phyllids have a simple structure, consisting primarily of a single cell layer with a central, multilayered vein—the midrib (*5*). They originate from a single phyllid initial cell that undergoes multiple rounds of oblique divisions, followed by further divisions occurring in its daughter cells (*6*). Despite their immense morphological diversity and the anatomical differences between bryophyte phyllids and vascular plant leaves, they typically exhibit a laminar structure and determined growth—key adaptations for optimizing light capture and ensuring a similar physiological function.

The molecular basis and cellular dynamics of leaf development have been primarily researched in flowering plants (*7*, *8*). Studies across diverse species suggest that, despite differences in final morphology, leaf formation follows a shared developmental program that includes common growth dynamics and regulatory mechanisms (*9*–*12*). For instance, most leaves exhibit gradients of cellular growth and divisions along their proximodistal axis, which are coupled with the progression of cell differentiation (*13*–*16*). Tuning this common developmental regulation is crucial for the establishment of the final shape and size of the organ (*12*, *17*–*19*). While the mechanisms behind these cellular gradients remain unclear, computational modeling and surgical experiments suggest that they may be controlled by unknown intrinsic signal likely diffusing from the base of the organ and creating concentration gradient along its main axis (*12*, *14*, *16*, *17*). These gradients of cell growth and differentiation are modulated by various molecular players such as TCP proteins and microRNAs (*11*, *15*, *20*), but they may also be, at least partially, tuned by plant hormones (*11*, *12*, *19*–*21*).

Auxin plays a particularly important role in regulating developmental gradients, as it controls cell division, elongation, and differentiation in a dose-, position-, and context-dependent manner (*22*). In angiosperm leaves, local auxin accumulation, driven by polar auxin transport, triggers organ initiation and outgrowth (*23*–*25*). Auxin maxima at the leaf tip and margin have been proposed to function as global or local organizers, coordinating growth within the developing organ (*12*, *17*, *26*–*28*). Subsequently, auxin spreads basipetally from the tip to the base through the leaf epidermis and margin, correlating with the progression of cell differentiation (*19*, *28*, *29*).

Unlike flowering plants, moss phyllid development is thought to be regulated primarily by a cell lineage–dependent mechanism (*6*, *30*). However, cell divisions in the phyllid persist longest at its base (*6*, *31*, *32*), suggesting that positional cues might also contribute to phyllid organogenesis. Despite the independent evolution origins of moss phyllids and angiosperm leaves, the core genetic components involved in auxin biosynthesis, transport, and signaling are conserved across both lineages, indicating that auxin might provide positional information during phyllid development (*33*–*38*). However, the spatial and temporal cellular dynamics underpinning phyllid organogenesis remain poorly understood, and it is unknown whether auxin regulates growth and differentiation gradients in this context.

Here, we address these open questions in the model moss *P. patens* through a novel time-lapse imaging protocol combined with pharmacological, genetic, and computational approaches. Specifically, we aim to test whether the core morphogenetic principles governing leaf development in flowering plants are shared with bryophytes and contribute to phyllid morphogenesis.

## RESULTS

### Positional cues control growth and cell division during phyllid morphogenesis

**Fig. 1.**
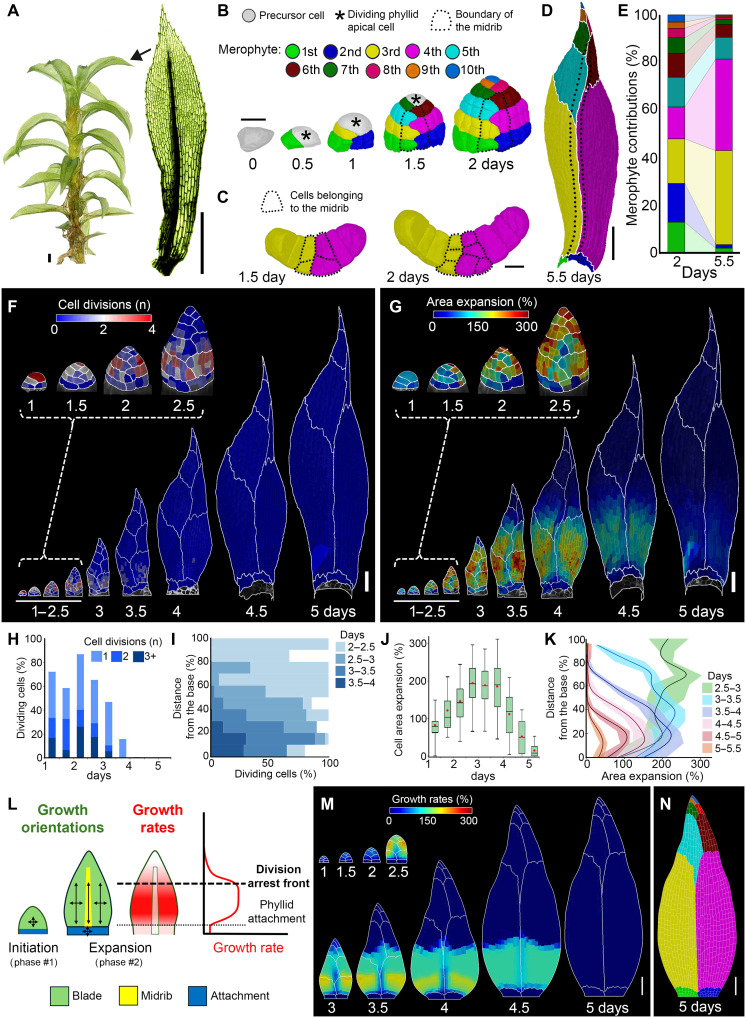
Positional cues control growth and cell division during phyllid morphogenesis. (**A**) *P. patens* gametophore isolated from a 1-month-old colony and the representative upper phyllid with visible midrib. (**B**) 3D lineage tracing of the upper phyllid initiation. Colors represent merophytes; dotted lines, midrib; asterisks, dividing apical cell. (**C**) Cross section of the third and fourth merophytes. Dotted lines mark midrib cells. (**D**) Fully developed upper phyllid with colors marking merophytes. (**E**) Relative contribution of different merophytes from 2 days to the final size of phyllid (5.5 days). (**F** and **G**) Heatmaps of cell divisions (F) and area expansion (G) for the upper phyllid. Values are displayed at the earlier time point. (**H** to **K**) Quantification of cell divisions (H) and cell area expansion (J) and their distribution along the normalized distance from the organ base [(I) and (K)]. The shades contain the second and third quartiles, and the lines indicate the median (*n* = 18, 46, 91, 226, 490, 799, 931, 939, and 938 cells at consecutive time points; three time-lapse series). (**L**) Assumptions of the model of upper phyllid development. Growth is slow and relatively isotropic at initiation (phase #1). Subsequent growth is homogeneous and anisotropic in the phyllid blade, very anisotropic in the midrib, and isotropic in the attachment zone (phase #2). Growth rates depend on tissue type and differentiation. Cells stop dividing and differentiate when passing beyond a given distance (thick dotted line) from the phyllid base. Following differentiation (phase #3), cells stop dividing, elongate, and progressively stop growing. (**M**) Model output colored by areal specified growth rate and merophytes marked with white lines. (**N**) Resultant distribution of merophytes. Scale bars, 500 μm (A), 20 μm [(C) and (D)], 200 μm (E), and 100 μm [(G) and (H) and (N) and (O)]. See also fig. S1 and movies S1 to S3.

The mature upper *Physcomitrium* phyllid is a small (about 2-mm-long) laminar organ with a lanceolate shape that is slightly folded along its central midrib (Fig. 1A). Its initiation and early development occur at the apex of the leafy shoot from a single initial cell that is tightly covered by older primordia and numerous axillary hairs. For these reasons, live imaging of the upper phyllid development has been considered too challenging (*6*), which hindered our understanding of the cellular dynamics underpinning its formation and expansion. To overcome these limitations, we developed dissection and imaging methods allowing us to follow the development of the entire organ from a single initial cell until the phyllid reaches its full size, 5 to 6 days after initiation (see Materials and Methods). Using MorphoGraphX software (*39*), we segmented all cells in two-dimensional (2D) or 3D for all time points, computed cellular growth rates and orientations, tracked cell divisions, and obtained lineage information (Fig. 1, fig. S1, and movies S1 and S2).

As previously observed, the initiation of the phyllid started with alternating left-right oblique divisions of the precursor cell (Fig. 1B) (*6*, *36*). In the upper phyllid, these divisions consistently generated around 10 daughter cells (10 ± 1 cells, *n* = 23 primordia) that gave rise to a series of clonal sectors (merophytes) with a zigzagging boundary in the middle of the organ (Fig. 1, B, D, and E). This zigzagging pattern subsequently straightens as strong elongation of the midrib aligns initially oblique sector boundaries with the main (longitudinal) organ axis (Fig. 1F). The first rounds of subsequent divisions of the daughter cells (from 1 to 2 days) were always longitudinal (oriented parallel to the main axis of the organ) and produced additional cell files in the basal merophytes (Fig. 1B). Subsequently, a single cell in each sector, located in the middle of the primordium, divided longitudinally, giving rise to the midrib (Fig. 1, B and C). These predictable patterns of precursor cell divisions, as well as a consistent number of merophytes, support the idea that phyllid initiation is controlled by lineage-based cues (*6*).

We next evaluated whether merophytes could act as autonomous domains, inheriting developmental instructions from their initials as previously suggested (*6*). To investigate this, we quantified cell growth and divisions throughout phyllid development (Fig. 1, F to K, and movie S1). During the initiation phase (0 to 2 days), cellular growth was relatively slow and uniform, while most cells continued dividing (Fig. 1, F, H, and J). From day 2, cells in the primordium—except those at the phyllid base, which form attachments to the stem—accelerated their growth along the longitudinal axis of the organ (Fig. 1, G, J, and K; fig. S1, D to H; and movies S1 and S2). Shortly after midrib establishment (at 2 days), the mediolateral growth in this region became strongly restricted (Fig. 1G and fig. S1, D and E). From 2.5 days after initiation, cell divisions progressively decreased and a gradient emerged, with divisions gradually ceasing from the tip to the base (Fig. 1, F, H, and I). This was followed by a basipetal gradient of cellular growth, first visible at 3 to 3.5 days and persisting until the end of phyllid development (Fig. 1, G and K). Prolonged growth at the phyllid base resulted in a substantial size increase in the proximal sectors, with the third and fourth sectors contributing nearly 80% for the final organ size (Fig. 1, D and E). Contrary to expectations of inherited behavior from the phyllid initial cell, cell divisions and growth did not follow patterns that would be expected solely from clonal regulation within merophytes but formed a global gradient irrespective of sector boundaries (Fig. 1, F and G). This suggests that positional cues rather than sector-dependent lineage control phyllid development after initiation. In addition, both cell divisions and growth were maintained at a relatively fixed distance from the organ base (Fig. 1, F and G, and fig. S1, D and E), indicating a global regulation of organ morphology, possibly mediated by signals originating from the organ base. Together, our live-imaging data suggest that phyllid initiation is mainly driven by lineage-autonomous behavior, whereas subsequent development across sectors is primarily regulated by positional cues that spatially restrict cell divisions to the proximal organ region.

To test this hypothesis and determine whether the identified developmental rules were sufficient to account for phyllid shape, we integrated our quantitative observations into a simple 2D computational model of phyllid development. In our model, cells are represented as polygons and can individually control their growth and cell division depending on their location within the phyllid. The simulation followed three distinct phases of phyllid development (Fig. 1L). In phase #1 or phyllid initiation (0 to 1.5 days), the primordium is divided into an upper zone and a phyllid attachment zone that is composed of undifferentiated cells growing slowly and isotropically. In phase #2 or phyllid expansion (2 to 4 days), cell division and growth accelerate in the upper zone of the phyllid, while the phyllid attachment zone grows very slowly. The midrib is introduced with reduced growth centrally along the mediolateral organ axis. In phase #3 or phyllid maturation (after 4 days), all cell divisions cease, and the phyllid progressively stops expanding. The main assumptions of the model are as follows (see table S1 for model parameters): (i) Cells have positional information and know their location between the base of the phyllid and the apical cell. This creates a polarity field. (ii) The apical cell divides obliquely in an alternating left-right pattern, and daughter cell divisions follow the shortest of the paths parallel or perpendicular to the polarity field. (iii) Undifferentiated, dividing cells grow relatively fast. (iv) Cells divide upon reaching a predetermined size threshold. (v) Beyond a given distance from the organ base, cells stop dividing and differentiate. (vi) Differentiating cells stop dividing, elongate transiently before gradually ceasing growth as observed in time-lapse data (fig. S1, A to C). In the model, cells decide their own growth rates depending on their location in the phyllid, and the growth rate is specified at the cellular level.

Although the 2D model includes only a minimal set of roles, we found that it could qualitatively recapitulate the growth dynamics of developing phyllids by using positional cues to control growth and cell division. This resulted in the patterning of sectors, including their relative size and distribution, comparable to those observed experimentally (Fig. 1, D, F, G, M, and N, and movie S3). This indicates that the minimal set of assumptions (table S1) is sufficient to explain how phyllids achieve their final shape. Furthermore, this result supports the hypothesis that positional information controls phyllid development.

### Auxin acts as a positional cue, with PINs reducing intracellular auxin concentration to shape phyllid development

Global growth and cell division gradients appear to govern phyllid development, but the nature of underlying signals through which they are controlled remains unclear. During vascular plant organogenesis, auxin plays a critical role in providing positional information for the establishment of tissue patterns, organ polarity, and the coordination of cell differentiation and growth (*12*, *19*, *21*–*28*, *40*). Moreover, alterations in auxin levels or transport affect phyllid shape and size in *Physcomitrium* (*34*–*37*, *41*), suggesting that this phytohormone may be crucial for regulating gradients of cell division and growth during phyllid morphogenesis.

**Fig. 2.**
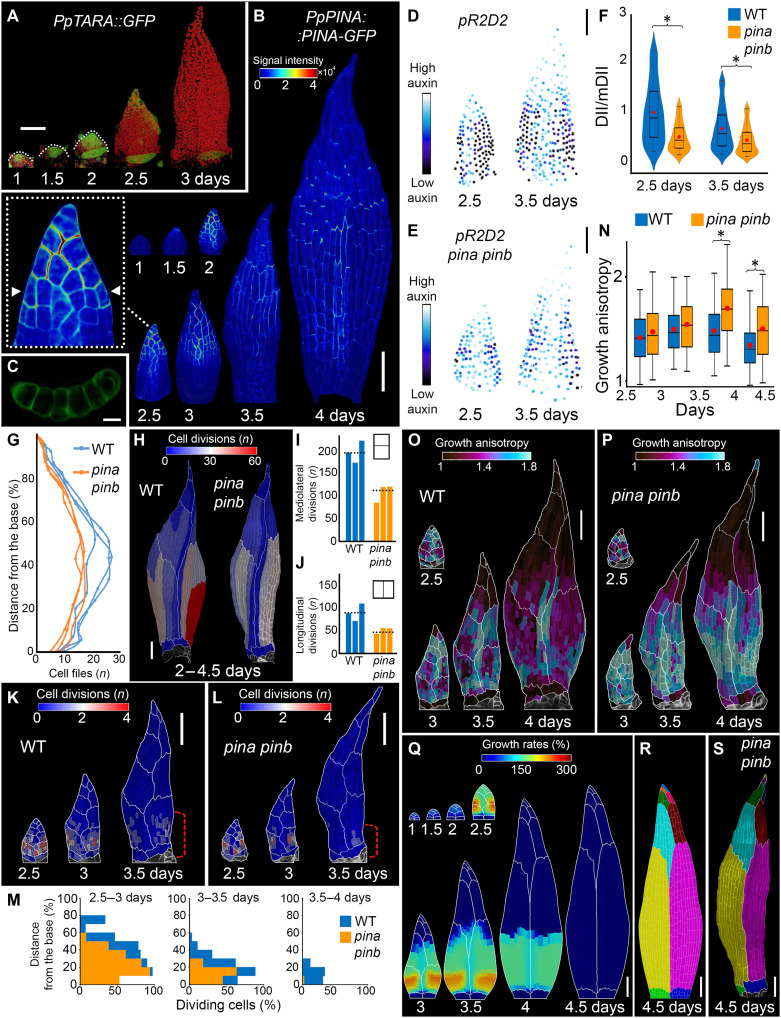
Auxin acts as a positional cue, with PINs reducing intracellular auxin concentration to shape phyllid development. (**A**) Localization of *PpTARA::GFP* (green) expression in the upper phyllid*.* Chloroplast fluorescence in red. (**B** and **C**) Localization of *PpPINA::PINA-GFP* expression in the upper phyllid. Heatmap represents GFP intensity. Inset: close-up view of the phyllid. Arrowheads: the position of the cross section shown in (C). (**D** and **E**) Segmented nuclei in phyllids at 2.5 and 3.5 days, color-coded by auxin sensing level (DII/mDII signal ratio). (**F**) Quantification of DII/mDII signal ratio (*n* = 3 phyllids for WT and *pina pinb* at 2.5 days and *n* = 5 phyllids at 3.5 days). Asterisks indicate statistical significance (*P* < 0.05, *t* test). (**G**) Phyllid width (cell files) as a function of the normalized distance from its base. (**H**) Heatmaps of cumulated cell divisions (2 to 4.5 days) for WT and *pina pinb* phyllids. (**I** and **J**) Quantification of mediolateral (I) and longitudinal (J) cell divisions (2–4.5 days). Dotted lines: average values from three time-lapse series. (**K** and **L**) Heatmaps of cell divisions in WT (K) and *pina pinb* (L) phyllids. Red brackets: the extent of the cell division zone. (**M**) Number of dividing cells as a function of the normalized distance from the base. (**N**) Growth anisotropy in WT and *pina pinb* (*n* = 3 time-lapse series, *P* < 0.05, *t* test). (**O** and **P**) Heatmaps of growth anisotropy in the WT (O) and *pina pinb* (P). (**Q** and **R**) Model of the *pina pinb* mutant phyllid with reduced cell divisions and increased growth anisotropy compared to WT. Model output colored by areal specified growth rate (Q). Resultant distribution of merophytes (R). (**S**) Upper phyllid of *pina pinb* mutant with merophytes highlighed. Scale bars, 100 μm [(B), (D) and (E), (H), (K) and (L), (O) and (P), and (Q) to (S)], 10 μm (C), and 20 μm (A). See also figs. S2 to S7 and movies S4 to S7.

To investigate whether the role of auxin as a positional cue is conserved in moss organogenesis, we first monitored the dynamics of auxin biosynthesis gene expression and auxin sensing during upper phyllid development, both spatially and temporally. In *P. patens*, auxin is mainly synthesized through the tryptophan-dependent pathway controlled by *TRYPTOPHAN AMINO-TRANSFERASE* (*TAR*) and *YUCCA* (*YUC*) genes (*42*, *43*). Published datasets show that *TARA*, *TARC*, and *YUCF* genes are the most highly expressed in phyllids (fig. S2), consistent with previous reports of similar promoter activity for *TARA* and *TARC* (*44*). We detected a clear green fluorescent protein (GFP) signal in phyllids of the *TARA* promoter line, but not in the *YUCF* promoter line. The most parsimonious explanation is that *YUC* expression remains below our reporter’s detection threshold. Because the conversion of tryptophan (TRP) to IPyA by TAR enzymes is a limiting step in auxin biosynthesis in *Physcomitrium* (*42*), we reasoned that a *TAR-GFP* reporter would provide a suitable proxy for identifying putative auxin biosynthesis sites in phyllids. The promoter activity of *TARA* was low on day 1, peaked on day 2, and rapidly declined to undetectable levels by day 3 after upper phyllid initiation (Fig. 2A). *TARA* promoter activity was first observed in the apical cell and then mainly detected in a few cells at the primordium tip and distal margin (Fig. 2A). These data suggest that auxin is likely produced at the organ tip during the early developmental stages of the upper phyllid. Consistently, auxin sensing monitored by the ratiometric biosensor *R2D2* was first detected in the distal part of the upper phyllid and subsequently spread toward its base (Fig. 2, D and F, and fig. S3C), in alignment with a previous report (*45*). Together, these data indicate that auxin moves throughout the organ from its source cells at the phyllid tip toward its base.

In angiosperms, the spatial distribution of auxin within tissues is primarily governed by auxin efflux carriers belonging to the *PIN-FORMED* (*PIN*) family, which are responsible for polar auxin transport (*23*–*25*, *46*, *47*). The role of PIN proteins in exporting auxin out of cells is conserved in *Physcomitrium* (*35*, *48*), whose genome encodes three canonical *PIN* genes: *PINA*, *PINB*, and *PINC*. All three genes are expressed in phyllids with both *PINA* and *PINB* genes detected at similar levels, significantly higher than *PINC* (*34*, *38*, *49*, *50*). While phyllid development is only mildly affected in single mutants, it is disrupted in *pina pinb* double mutant that develops narrower organs, indicating that PINA and PINB act redundantly to control phyllid morphogenesis (*32*, *38*). To understand the role of efflux carriers in the dynamics of auxin transport, we precisely monitored the distribution of PINA and PINB expression during upper phyllid development. PINA-GFP was first detected in the apical cell 1.5 days after organ initiation, and its expression domain subsequently expanded toward the more proximal regions of the phyllid, invading up to two-thirds of its length (Fig. 2B). PINB-GFP expression patterns and timing were overall similar to PINA-GFP (fig. S3, A and B). The basipetal spread of the expression of these transporters and accompanying auxin sensing (*R2D2*) correlated with the progression of the cell division arrest front (Figs. 1G and 2K). This suggests that PINs may mediate a basipetal auxin movement to regulate phyllid development through auxin-mediated inhibition of cell divisions.

If this hypothesis is correct, then eliminating PIN activity should limit the progression of cell division arrest and consequently expand the division zone toward the phyllid tip as auxin would no longer travel as far as in the wild type (WT) from its source at the phyllid tip to inhibit divisions. To test this hypothesis, we introduced a plasma membrane marker in a *pina pinb* double mutant (*34*) and monitored the development of the upper phyllid with time-lapse imaging. The *pina pinb* mutant phyllids were composed of fewer cell files than WT phyllids, in agreement with previous studies (Fig. 2G) (*32*). Cell divisions were reduced in both longitudinal (−51%, on average) and mediolateral (−43%, on average) orientations (Fig. 2, H to J). However, contrary to our expectations, while the general shape of basipetal gradients of cell division were comparable to the WT, the division zone was spatially reduced to more proximal regions of the phyllid in the mutant (Fig. 2, K to M; fig. S4; and movie S4). The duration of cell divisions was also shortened in the absence of auxin efflux carriers (Fig. 2M; fig. S4, A and C; and movie S4). These observations raised the hypothesis that the lack of *PINA* and *PINB* could promote auxin diffusion through the organ by increasing its intracellular concentration, instead of restricting it. To test this, we knocked-out *PINA* and *PINB* in the transgenic line expressing the *R2D2* biosensor using CRISPR-Cas9–mediated gene editing and recovered mutant lines displaying phyllid shape defects similar to those previously reported (fig. S5). Consistently, we observed a significantly lower DII/mDII signal ratio in the *pina pinb* mutant compared to WT, reflecting an overall increase of auxin sensing levels throughout phyllids (Fig. 2, D to F, and fig. S3C).

To explain these observations, we closely examined the subcellular localization pattern of PINA and PINB in growing phyllids. During the early stages of phyllid development, both PINA-GFP and PINB-GFP signals were primarily detected at membranes, without any clear basal polarization (inset in Fig. 2B and fig. S3, A and B). The previously reported polar or bipolar localization at the apical and basal membranes (*35*) was only observed in differentiated, nongrowing cells at the tips of the phyllids, starting from 3.5 days after initiation (Fig. 2B). Intriguingly, we found that a portion of PINA and PINB proteins was localized at membranes facing the extracellular space, suggesting that PINs might export auxin from the cytoplasm to the cell wall and potentially out of the organ (Fig. 2C and fig. S3B). Together, these findings indicate that, in contrast to vascular plants, plasma membrane localized PINs in moss may not participate in basipetal auxin movement during early phyllid development but rather reduce an overall intracellular auxin levels. In their absence, auxin could instead diffuse between cells via plasmodesmata, a scenario consistent with the presence of plasmodesmata in anticlinal walls observed during early phyllid development (fig. S6) (*29*, *51*–*56*).

Next, to test whether the inhibition of cell division affects organ shape as observed in *pina pinb* phyllid (Fig. 2, G and H), we modified the parameterization of our computational model of the upper phyllid (Fig. 1, M and N) by reducing the distance from the base at which cells are allowed to proliferate and accelerating the transition from phase #2 to phase #3, when all cell divisions cease. We did not aim to explicitly model auxin transport, but rather to evaluate whether auxin-induced changes in cell behavior could account for observed changes in phyllid morphology. These modifications reflect the observed higher auxin spread from the organ tip in the *pina pinb* mutant and assume that elevated auxin levels can override the influence of the basal zone, thereby forcing cells to differentiate earlier. With these simple changes, the model produced a narrower phyllid, resembling that of the *pina pinb* mutant. Nevertheless, the simulated organ was shorter than experimentally observed, suggesting that auxin-mediated inhibition of cell division alone is not sufficient to fully account for the mutant phenotype (fig. S7 and movie S5).

Apart from accelerating the transition from cell division to differentiation, auxin is also known to control cell elongation (*12*, *41*). We observed an increase in cellular growth anisotropy in the *pina pinb* double mutant (Fig. 2, N to P; fig. S8; and Movie S6). We tested the impact of this additional parameter by increasing the cell elongation rate in the model and found that the resulting simulated phyllid shape and sector distribution matched those observed in the *pina pinb* mutant (Fig. 2, Q to S, and movie S7). Together, our experiments and simulations show that auxin can fine-tune phyllid shape by modulating the underlying gradients through the inhibition of cell division and promotion of cell elongation.

### Auxin can disrupt cell division and growth gradients during phyllid development

Auxin appears to be crucial for phyllid morphogenesis by promoting the cessation of cell divisions and stimulating cell elongation, thereby influencing the organ’s final shape. When the auxin concentration is increased—such as through the removal of PIN proteins—higher auxin reduces the proliferative activity (Fig. 2, K to M) and increases growth anisotropy (Fig. 2, N to P). This suggests that auxin is a major regulatory cue, sufficient to counteract intrinsic basal gradients that would otherwise sustain cell division at the phyllid base. To investigate this further, we sought to test whether auxin had the capacity to globally alter phyllid development by reprogramming cell behavior across the entire organ. We therefore hypothesized that supplying exogenous auxin should lead to a rapid cessation of cell divisions and enhanced cell elongation throughout the primordium, ultimately producing a narrow phyllid composed of fewer, elongated cells.

**Fig. 3.**
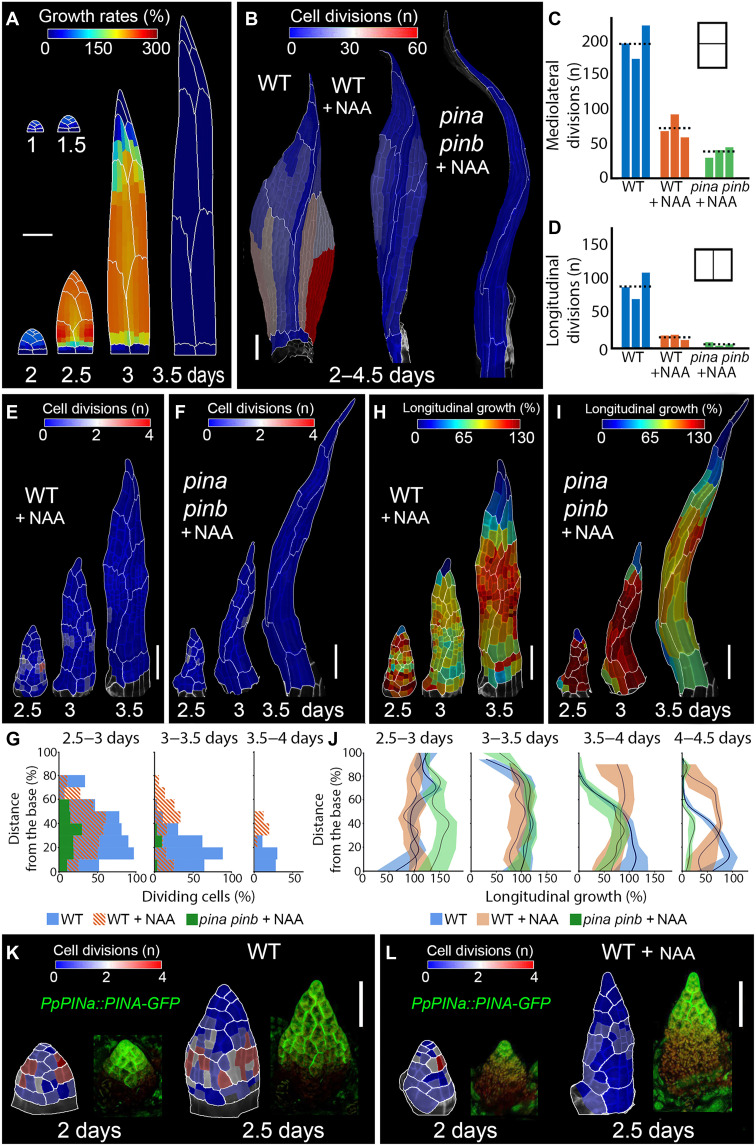
Auxin can disrupt the basipetal gradients during phyllid development. (**A**) Model of the phyllid with cell divisions eliminated after phase #1 and an increased growth anisotropy. Model output colored by areal specified growth rate. (**B**) Heatmaps of cumulated cell divisions (2 to 4.5 days) for WT and *pina pinb* mutants upper phyllids treated with auxin. (**C** and **D**) Quantification of mediolateral (C) and longitudinal (D) cell divisions (2–4.5 days) in WT and *pina pinb* phyllids treated with auxin (three time-lapse series). Dotted lines show average values. (**E** and **F**) Heatmaps of cell divisions in WT (E) and *pina pinb* mutant (F) treated with auxin. (**G**) Quantification of the number of dividing cells as a function of the normalized distance from the phyllid base. (**H** and **I**) Heatmaps of longitudinal growth in WT (H) and *pina pinb* mutant phyllids (I) treated with auxin. (**J**) Quantification of the longitudinal growth as a function of the normalized distance from the phyllid base (226, 490, 799, and 931 cells for WT; 193, 271, 321, and 325 cells for WT treated with auxin; and 201, 234, 239, and 237 cells for *pina pinb* treated with auxin; three time-lapse series). Heat values are displayed at the earlier point. (**K** and **L**) Localization of cell divisions and of the expression of *PpPINA::PINA-GFP* at early developmental stages of upper phyllids in the WT (K) and WT treated with auxin (L). Heatmaps of cell divisions (left) and PINA-GFP localization (right). GFP in green and autofluorescence in red. Scale bars, 100 μm [(A) and (B), (E) and (F), and (H) and (I)] and 50 μm [(K) and (L)]. See also figs. S8 to S12 and movies S8 to S12.

To explore this idea, we first used our model to simulate the growth of the upper phyllid under overall elevated auxin levels. Specifically, the transition from phase #2 to phase #3 was accelerated (i.e., faster cessation of cell divisions), and cell elongation was increased compared with the WT phyllid simulations (table S1). Under these assumptions, our model produced a very narrow, elongated phyllid (Fig. 3A and movie S8).

To validate the model’s predictions, we treated 1-day-old upper phyllid primordia with 1-naphthaleneacetic acid (NAA), a PIN-transportable synthetic auxin, and tracked their subsequent development with time-lapse imaging. NAA treatment significantly altered phyllid morphology. As expected, cell divisions and mediolateral growth were reduced, while longitudinal growth rates increased, producing much narrower organs compared to both the wild-type and the *pina pinb* mutant (Fig. 3, B to D; and figs. S9, A, C, and E and 10). Unexpectedly, however, cell divisions did not cease uniformly across the organ. Instead, active cell division persisted in the central region of the phyllid, whereas the basal region—which typically exhibits high division rates—stopped dividing earlier (Fig. 3, E and G; fig. S9, A, C, and E; and movie S9). The typical growth gradient was also disrupted, with cell growth lasting longer in the middle than at the base (Fig. 3, H and J; figs. S6, B, D, and F and S8; and movies S9 and S10). The sustained mediolateral growth in the organ’s center (fig. S8 and movie S10) ultimately led to the formation of a lanceolate phyllid, but not a very narrow organ as predicted by our model (Fig. 3, A and B).

This raised the question of why cell divisions and growth continued in the middle region of NAA-treated phyllids. Our data suggested that intracellular auxin levels decrease due to the activity of PIN proteins (Fig. 2, D to F). We therefore hypothesized that regional differences in PIN expression could determine how cells respond to exogenous auxin. In WT plants, PINA expression was not only restricted to the organ tip—where cells elongate and differentiate—but also partially overlapped with the distal region of the division zone (Figs. 2B and 3K). Instead, PINA expression was absent at the phyllid base, where most cell divisions take place (Fig. 2B and 3K). Upon NAA treatment, we observed that this apical-basal distribution pattern remained largely unchanged (Fig. 3L and fig. S11). From these observations, we speculated that when exogenous auxin is applied, cells located at the phyllid base are likely to experience higher auxin levels and thus cease dividing first. Conversely, in the upper portion of the proliferative zone (adjacent to or overlapping with PINA expression), PIN-mediated auxin efflux may prevent cells from reaching the auxin threshold required for immediate differentiation.

If this is correct, then external auxin application in the absence of PINs should further eliminate the divisions in this middle zone. Consistent with this hypothesis, treating the *pina pinb* mutant with NAA caused cell divisions to cease very early and largely abolished the sustained divisions observed in the central region of auxin-treated WT phyllids (Fig. 3, B to G; fig. S12; and movie S11). In addition, longitudinal growth was further enhanced, while mediolateral growth reduced, effectively canceling the inverted growth gradient observed in the NAA-treated WT (Fig. 3, H to J; fig. S13; and movie S12). As a result, a very narrow, rod-like phyllid developed, resembling the model outcome predicted for auxin-treated phyllids (Fig. 3A).

Together, these data show that auxin is sufficient to disrupt intrinsic global gradients of cell division and growth. This observation further supports the idea that PINs in moss can locally lower auxin concentration to regulate the switch from cell divisions to elongation. This result highlights the importance of PIN-driven auxin homeostasis in shaping phyllid development, underscoring a complex interplay between global auxin gradients and localized PIN activity in determining the spatial patterns of cell division and elongation.

### A temporal shift in auxin-triggered cell differentiation accounts for the juvenile-to-adult phyllid shape transition

**Fig. 4.**
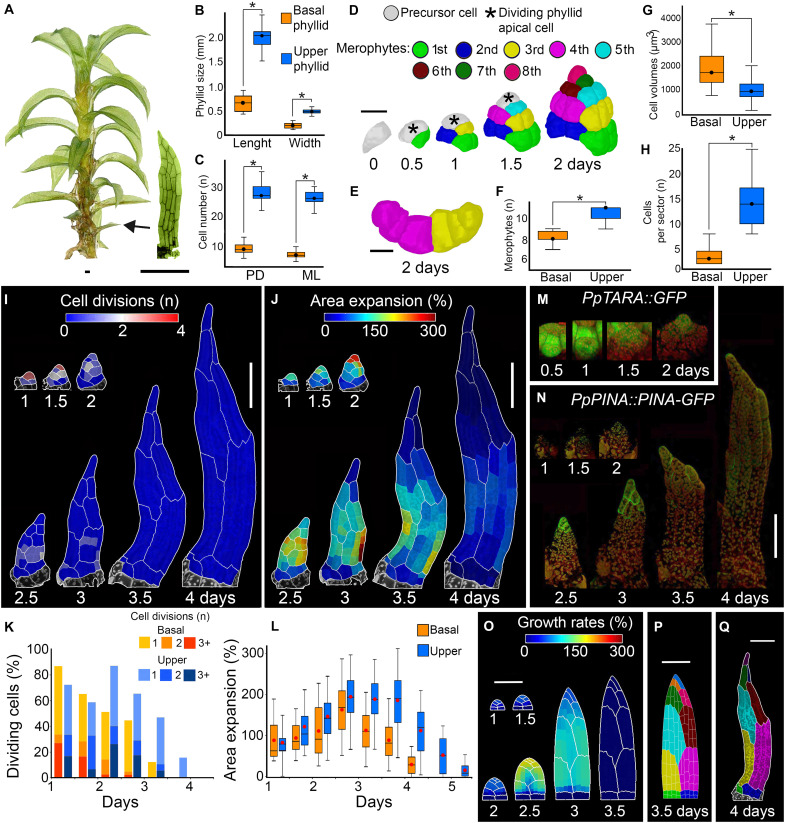
A temporal shift in auxin-triggered cell differentiation accounts for the juvenile-to-adult phyllid shape transition. (**A**) *P. patens* gametophore isolated from a 1-month-old colony and a representative basal phyllid. (**B** and **C**) Quantification of basal (juvenile) and upper (adult) phyllid size (B) and cell number (C) along proximodistal (PD) and mediolateral (ML) organ axes (*n* = 21 basal and 35 upper phyllids). (**D**) 3D lineage tracing of the basal phyllid initiation. Colors, merophytes; asterisks, dividing apical cells. (**E**) Cross section of the third and fourth merophytes. (**F** to **H**) Quantification of the number of merophytes per organ (F) (*n* = 26 basal and upper phyllids); cell volumes in third, fourth, and fifth merophytes (*n* = 4 basal and upper phyllids) (G); and number of cells per major merophyte at 2 days (*n* = 12 basal and upper phyllids) (H). (**I** and **J**) Heatmaps of cell divisions (I) and area expansion (J) for the basal phyllid. Values displayed at the earlier time point. (**K** and **L**) Quantification of cell divisions (K) and cell area expansion (L) (*n* = 15, 43, 94, 158, 238, 201, and 205 cells at consecutive points for the basal phyllid and 18, 46, 91, 226, 490, 799, 939, and 938 for the upper phyllid; three time-lapse series). (**M** and **N**) Localization of *PpTARA::GFP* (M) and *PpPINA::PINA-GFP* (N) activity in the basal phyllid. GFP in green, chloroplasts in red. (**O** and **P**) Model of the basal phyllid where cell division stops earlier as compared to the upper phyllid. Model output colored by areal specified growth rate (O). Resultant merophyte distribution (P). (**Q**) Basal phyllid with merophytes highlighted. Asterisk indicates significance (*P* < 0.05, *t* test). Scale bars, 500 μm (A), 20 μm [(D) and (F)], and 100 μm [(I) and (J) and (O) to (Q)]. See also figs. S13 to S15 and movies S13 to S15.

Phyllid morphology changes gradually during *Physcomitrium* gametophore ontogeny (Fig. 4A) (*31*, *32*), a process known as heteroblastic development (*57*). Basal (juvenile) phyllids lack a midrib and are substantially smaller, containing significantly fewer cells than upper phyllids (Fig. 4, A to C). They consistently maintain a narrow width, except at the tapering tip (Fig. 4A). This narrow morphology with a reduced cell number, mirrors that of auxin-treated upper (adult) phyllids, especially in the *pina pinb* mutant (Fig. 3B), suggesting that auxin could play a role in the juvenile-to-adult phase transition in moss.

To investigate this, we first analyzed when the initial differences between basal and upper phyllids were established. Although the alternating left-to-right oblique divisions of the precursor cell were observed in both basal and upper organs (Figs. 1B and 4D), the apical cell produced significantly fewer merophytes in the basal phyllid (8 ± 1 versus 10 ± 1 cells) (Fig. 4, D to F). Merophytes also consisted of fewer but bigger cells as early as 2 days after initiation, and longitudinal divisions were completely absent in the basal phyllid (Fig. 1C and 4, F to H). In addition, cell divisions and cell growth stopped around 1 day earlier in the basal phyllid (Fig. 4, I to L, and movie S13) as compared to the upper phyllid (Fig. 1, F to G, and movie S1). This timing was similar to what we observed upon auxin treatment of the upper phyllid in the *pina pinb* mutant (Fig. 3, F and G). Although basipetal gradients of cell division and expansion were present in the basal phyllid, they persisted very briefly (Fig. 4, I and J, and movie S13). After initiation, the longitudinal growth was comparable to that observed in the upper phyllid, while the mediolateral growth was strongly restricted (fig. S14 and movie S14). Together, this indicates that a very early onset of cell differentiation in the basal phyllid, rather than changes in the basipetal cell division gradient shape, controls heteroblastic development in the moss.

To test the role of auxin in driving this early shift from cell division to differentiation, we first examined auxin biosynthesis in the basal phyllid using a *TARA* promoter reporter line (*43*, *44*). Unlike in the upper phyllid, where *TARA* expression peaked at 2 days postinitiation (Fig. 2A), the basal phyllid showed a strong *TARA* signal in the initial cell at 0.5 days, which gradually diminished until 2 days after initiation (Fig. 4M). This finding suggests that auxin levels might be higher during the very early stages of basal phyllid outgrowth compared with the upper phyllid. In addition, both PINA-GFP and PINB-GFP expressions in the basal phyllid were much weaker than in the upper phyllid, appearing only in a few differentiating cells at the organ tip from 2.5 days postinitiation before fading rapidly (Fig. 4N and fig. S15), which indicated a reduced auxin export capacity. Unfortunately, technical limitations prevented us from obtaining reliable R2D2 signal measurements in developing basal phyllids. Collectively, these observations support the idea that an early, auxin-triggered transition from cell division to differentiation may underlie basal phyllid morphology.

We tested this further using model simulations. Specifically, we modified our initial model of upper phyllid development (Fig. 1, M and N) by accelerating the timing of cell division cessation (i.e., a faster transition from phase #2 to phase #3). With this simple change, the model simulated a smaller organ, similar in shape and size to the observed basal phyllid (Fig. 4O and movie S15). In contrast to the model of the upper phyllid, this simulation produced a narrow organ with merophytes located near its base contributing much less to overall organ size, which reflected our biological observations (compare Fig. 1, D and N with Fig. 4, P and Q). Together, these data indicate that a temporal shift in the auxin-driven basipetal cell differentiation gradient is likely sufficient to account for the shape changes characterizing the juvenile-to-adult phase transition, a defining feature of phyllid heteroblastic development.

## DISCUSSION

This study sought to elucidate the fundamental principles underlying phyllid morphogenesis in the moss *P. patens*, a model bryophyte. By applying innovative methods to track and quantify cellular growth from a single initial cell to a fully expanded organ, we characterized phyllid development at an unprecedented spatiotemporal resolution.

Our data confirm previous observations that lineage-based cues dictate the division patterns of apical cell derivatives during the onset of phyllid development (*6*). However, subsequent stages appear to be mainly regulated by global gradients of cell division, growth, and differentiation, independent of cell lineage. Cell divisions are maintained at a defined distance from the base of the organ and gradually cease following a basipetal wave of differentiation. Both upper and basal phyllids follow a similar developmental trajectory, but the smaller size of basal phyllids results from an earlier cessation of cell division and a premature transition to cell differentiation.

Overall, phyllid morphogenesis parallels key aspects of leaf development previously described in *Arabidopsis thaliana*. While early *Arabidopsis* leaf patterning is guided by positional information—in contrast to moss phyllids—later developmental stages feature a basipetal gradient of cell division and growth, with marked differences in the timing of gradient establishment in juvenile and adult organs in both plant lineages (*6*, *12*, *16*, *18*, *19*). Moreover, cell division and growth zones are restricted to the organ base, suggesting a conserved role for basal intrinsic cues in patterning these zones. Consistent with our findings in moss phyllids, a model of leaf morphogenesis supports a key role for basally derived signals, although their molecular nature remains unclear (*12*, *16*, *17*). Beyond *Arabidopsis*, comparative morphometric analyses across eudicots indicate that similar leaf shapes can be attained through diverse late growth patterns. While the early phase of cell division is generally conserved, the directionality of differentiation may vary, with some species exhibiting acropetal or bidirectional waves, or a more even distribution of both divisions and differentiation (*15*).

**Fig. 5.**
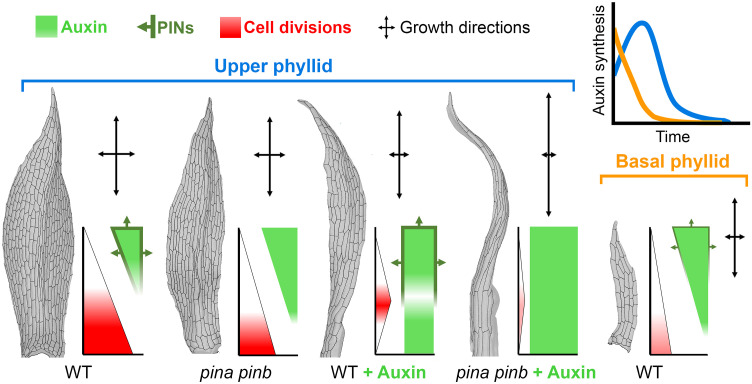
Putative model for auxin regulation of phyllid shape. By default, cell divisions are restricted to the organ base while auxin is produced at the tip and diffuses basipetally. Auxin inhibits cell divisions and promotes cell differentiation and elongation. In the WT upper phyllid, the activity of PIN auxin exporters maintains relatively low intracellular auxin levels, thereby allowing cell divisions to occur both relatively far from the organ base and over a longer period to produce broad phyllid. Eliminating PIN transporters raises intracellular auxin levels, enabling auxin to travel further away from the tip; consequently, cell divisions are reduced, and cell elongation is stimulated, yielding a narrower organ. External auxin application in the WT overrides the basipetal cell division gradient. Cells at the base, which never express PINs, respond strongly to the exogenous auxin by undergoing early differentiation and elongation, while cells juxtaposed to the PIN-expressing region keep dividing for some time, ultimately yielding an even narrower phyllid. In the absence of PINs, auxin treatment quickly suppresses cell divisions, triggering strong elongation across the entire phyllid and yielding to the formation of rod-shaped organs. In the basal phyllid, auxin production peaks during initiation, causing an early transition from cell division to cell differentiation, and ultimately, the development of a small organ with few cells.

In angiosperm organs, developmental gradients are closely linked to the dynamics of the phytohormone auxin. For instance, auxin promotes cell differentiation and growth in leaves (*11*, *12*), and the spatial distribution of auxin responses and transporters correlate with the progression of cell differentiation in both leaves and floral organs (*19*, *29*, *58*, *59*). Through genetic perturbations and pharmacological assays, we provide multiple lines of evidence that auxin plays similar roles in *Physcomitrium* phyllid development. Specifically, auxin likely synthesized at the phyllid tip serves as a spatiotemporal regulator of global gradients of cell division and growth, and it specifically promotes the transition from cell division to cell elongation and differentiation (*44*). By locally reducing intracellular auxin levels, evolutionarily conserved plasma membrane–localized PIN proteins fine-tune phyllid shape and size (*34*, *35*). Under normal conditions, auxin produced at the upper phyllid tip moves basipetally to induce cell differentiation, while cell divisions remain restricted to the organ base. PINA and PINB are frequently present at the membranes facing the outside of the organ in both phyllid tip, margin, and midrib (Fig. 2C and figs. S3, A and B and S15). This localization is never observed in *Arabidopsis* where PINs are always present at the membranes facing neighboring cells [e.g., (*12*, *24*, *25*)]. This PIN polarization in *Physcomitrium* strongly reduces intracellular auxin concentration by pumping a portion of auxin out of the organ, permitting sustained cell divisions at a distance from the base (Fig. 5). When PIN function is abolished, auxin accumulates intracellularly (Fig. 2E). This decreases cell divisions and stimulates cell elongation and differentiation, resulting in narrower phyllids compared to WT (Fig. 5). Exogenous auxin applications further inhibit cell divisions and stimulate cell elongation (especially in the absence of PINA and PINB), indicating that higher auxin concentrations in phyllids have increasingly stronger effect on their development, as observed in moss cultures grown with varying auxin concentrations (*41*).

Auxin levels in the midrib appear higher than in the phyllid blade (Fig. 2, D and E), which could suggest that auxin is involved in midrib formation and the midrib could transport auxin in *Physcomitrium*. In *Arabidopsis*, auxin is transported by polarized PIN efflux carriers (*46*), and PINs and auxin are upregulated in the developing prevascular tissue (*60*, *61*). While both PINA-GFP and PINB-GFP signals are detected in the moss midrib, in contrast to *Arabidopsis* PIN1, they are apolar and appear only 1.5 to 2 days after midrib development begins (Fig. 2B and fig. S4). In mutants lacking a midrib such as *lateral suppressor 1* (*pplas1*) and *pplas2*, the phyllid and cell morphology remains similar to WT (*62*, *63*). Together, these results suggest that the midrib plays only a minor role in auxin distribution throughout the phyllid and in determining final organ shape.

Exogenous auxin treatments further demonstrate the role of PINs in orchestrating gradients of cell growth and divisions. In WT phyllids, basal cells that lack PIN expression and are typically the last to differentiate, show a stronger response to auxin, and undergo premature elongation and differentiation (Fig. 5). This effect is enhanced in *pin* mutants, reinforcing the idea that PINs prevent cell differentiation by keeping intracellular auxin levels relatively low. Time-lapse imaging of basal phyllids supports this model, showing that early *TARA* activity and limited PIN expression likely contribute to their smaller size by triggering precocious differentiation compared to adult phyllids (Fig. 5).

Unlike in angiosperms, we propose that *Physcomitrium* PINs may not be involved in long-range polar auxin transport during early phyllid development, but rather in reducing intracellular auxin concentration by pumping auxin out of cells. This finding suggests that PIN function has diverged between angiosperms and mosses. The single PIN protein from the streptophyte alga *Klebsormidium flaccidum* is also distributed laterally and lacks specific polar localization when expressed in tobacco and moss cells (*64*). This finding has led to the proposal that PIN’s ancestral role may have been to export auxin into the environment. Consistently, most of the auxin in moss cultures appears to accumulate in the medium (*64*). Thus, *Physcomitrium* PIN localization patterns could reflect an ancestral state in the green lineage. Moreover, this implies that intracellular auxin movement in phyllids may rely on alternative mechanisms, such as plasmodesmata-mediated diffusion. Symplasmic auxin movement has been implicated in *Arabidopsis* root development, hypocotyl tropism, and leaf venation (*53*–*56*), and auxin has been also shown to move via plasmodesmata in *Arabidopsis* leaf epidermis (*29*). In *Physcomitrium*, symplasmic auxin movement may be sufficient to regulate branch patterning in leafy shoots (*52*, *53*), and numerous plasmodesmata are present in the developing phyllids in *Physcomitrium* (fig. S6). Further research is needed to determine whether this mechanism is sufficient alone to regulate developmental patterning in moss (*65*).

Another key difference between angiosperms and mosses is the degree to which auxin regulates organ development. In *Arabidopsis* leaves, exogenous auxin accelerates cell growth and cell differentiation but has limited effects on intrinsic developmental gradients (*12*, *28*). In contrast, exogenous auxin treatments in *Physcomitrium* can override global basipetal gradients of cell division and growth, and in *pin* mutants, this leads to a notable perturbation of organ development, exemplified by the formation of rod-shaped phyllids. Overall, this underscores the role of auxin in influencing the final phyllid shapes.

Together, our findings in *Physcomitrium* and previous work in *Arabidopsis* and other angiosperms reveal several shared principles of planar organ morphogenesis. First, organ development is characterized by an initial phase of cell divisions followed by a transition to cell expansion and differentiation, coordinated by global positional cues (*12*, *15*–*19*). Second, auxin serves as a key positional cue regulating the transition from cell division to differentiation, with distinct contributions in juvenile and adult organs (*19*). We conclude that the convergent evolution of angiosperm leaves and moss phyllids was driven by the repeated deployment of these deeply shared developmental principles, with lineage- and species-specific variations.

## MATERIALS AND METHODS

### Plant materials and growth conditions

The following *P. patens* transgenic lines were used: *PpEF1α::acyl-YFP* (this study), pina pinb double mutant (*34*), *pR2D2-1* (*45*) *PpPINApro::PpPINA-GFP* (*35*), and *PpTARA::GFP-GUS* (*44*). *PpEF1α::acyl-YFP* was transformed into a *pina pinb* double mutant. The *pina* and *pinb* mutations were introduced into the *pR2D2-1* transgenic line to achieve *pR2D2-1-pinapinb* using the CRISPR-Cas9. *EF1α::acyl-YFP*, *pina pinb*, and *PpPINApro::PpPINA-GFP* are in the Gransden background, and all other reporter lines are in the Reute background. Plants were cultivated in vitro in sterile conditions on BCDAT medium (*66*) with 0.7% agar in a growth chamber under continuous light (80 μmol m^−2^s^−1^) at 22°C with 60 to 70% relative humidity. Exceptionally, *PpR2D2-1* and *PpR2D2-1-pinapinb* were cultivated under a 16-hour light and 8-hour dark cycle.

### Construction of transgenes and plant transformation

Constructs for plant transformation were generated using standard cloning techniques. To construct *PpEF1α::acyl-YFP*, the acyl-YFP sequence (*67*) was first moved from the Gateway destination vector *pUBQ10::acyl-YFP* (*68*) to the *pENTR207* donor vector (Thermo Fisher Scientific) using BP Gateway reaction. The *acyl-YFP* sequence in *pENTRE207* vector was then moved to pT1OG moss transformation vector containing *PpEF1α* promoter (provided by K. Kosetsu, NIBB) and PTA1 homologous recombination sites using LR Gateway reaction to obtain *PpEF1α::acyl-YFP*. Final constructs were verified by sequencing and transformed into the wild-type using polyethylene glycol (PEG)–mediated homologous recombination method (*66*).

The *pina* and *pinb* mutations in the *R2D2-1* transgenic line (*45*) were generated using the Gateway based CRISPR-Cas9 vector system (*70*, *71*). Four protospacers (corresponding to single guide RNA) targeting the 5ʹ untranslated region (5ʹUTR) (position 198), the exon 1 (position 1086), the exon 4 (position 106), and the 3ʹUTR (position 140) of *PpPINA* (Pp3c23_10200V3.1) and four additional protospacers targeting the 5ʹUTR (position 205), the exon 1 (position 1023), the exon 4 (position 122), and the 3ʹUTR (position 203) of *PpPINB* (Pp3c24_2970V3.1) were designed using the CRISPOR software (*72*). For each targeted gene, the four complementary protospacer oligos (reverse and forward pairs) were annealed and then inserted downstream of an U6 promoter into the Bsa I site of the following *pENTR* vectors: *pENTR-PpU6P-sgRNA-L5L4*, *pENTR-PpU6P-sgRNA-L1R5*, *pENTR-PpU6P-sgRNA-R4R3*, and *pENTR-PpU6P-sgRNA-L3L2*. *PpR2D2-1* protoplasts were co-transformed with all the entry vectors that carry the *PpPINA* and *PpPINB sgRNAs* together with a modified *pZeo-Cas9-gate* destination vector that contains the Cas9 enzyme coding sequence. After antibiotic selection, the resistant clones were genotyped by polymerase chain reaction using primers external to the coding sequence of *PpPINA* and *PpPINB*. For *PpPINA*, the expected size for the endogenous locus size was of 3765 base pairs (bp), and we found large deletion (more than 1500 bp between Exon 1-1086 and 3ʹUTR-140 sgRNAs) for clones 25 and 29 using the primers P43 and P44. For *PpPINB*, the endogenous locus is of 3792 bp, and we also found large deletion in clones 25 and 29 (more than 3000 bp between 5ʹUTR-205 and 3ʹUTR-203 sgRNAs) using the primers P47 and P48.

### Gene expression data

*TAR* and *YUC* gene expression data were retrieved from the MAdLandExpression database (https://peatmoss.plantcode.cup.uni-freiburg.de/easy_gdb/index.php). These data were originally published in (*72*–*73*).

### Confocal microscopy

Zeiss LSM800 or LSM980 upright confocal microscope with diode lasers and 25× or 40× immersion objectives (1 numerical aperture, Apochromat) were used for imaging. Confocal z-stacks were acquired with 0.5- to 1-μm distance in z dimension at 1024 × 1024 for the young leaf primordium and 512 × 512 resolution for leaves at later developmental stages. Yellow fluorescent protein (YFP) from the membrane marker and green fluorescent protein (GFP) from the reporter lines were excited with a 488-nm diode laser, and the signal was collected at 400 to 614 nm and 500 to 550 nm, respectively. Autofluorescence was simultaneously collected at 656 to 700 nm.

Airyscan mode was used to detect the detailed localization of PpPINApro::PpPINA-GFP in the young upper phyllids. Samples were flipped, and images for both abaxial and adaxial sides were then merged together in MorphoGraphX software (*74*).

VENUS and TdTOMATO signals from *pR2D2-1* and *PpR2D2-1-pina pinb* reporter lines were collected as described before (*45*). Briefly, The *PpR2D2-1* and *PpR2D2-1* pina pinb was imaged with a Zeiss LSM980 confocal microscope and a water immersion objective lens of 25×. The signals of DII (TdTomato) and mDII (VENUS) were imaged sequentially to avoid cross-talks.

For time-lapse imaging of the upper phyllids, gametophores with newly emerged 12th phyllid were selected from plates 18 days old. The neighboring phyllids were gently removed with fine tweezers to expose the target phyllid for imaging. The same phyllid was imaged every 0.5 days for about 6 days until maturity. If necessary, then gametophores were tilted before the scanning to expose the abaxial side of the phyllid for imaging. Multiple overlapping stacks were taken to cover the samples exceeding the objective field of view. Plant samples were immersed in sterile water or chemical solutions during imaging. After each scanning, the plants were rinsed three times with sterile water, drained with tissue paper, and returned to the original growth condition in the growth chamber. A minimum of three independent timelapse series were acquired for each experiment.

### Auxin treatment

Auxin treatment was performed with 1 M 1-naphthaleneacetic acid (NAA, Sigma-Aldrich) solution. Plants were submerging samples each 12 hours in water with specified chemical solution during imaging. Treated samples were rinsed three times with sterile water after imaging and put back to the growth chamber.

### Electron microscopy

For plasmodesmata visualization, the samples were processed following a previously described procedure (*75*). Dissected phyllids at around 2.5 to 3.5 days after initiation were fixed overnight at 4°C in 2% glutaraldehyde and 2% formaldehyde (v/v) at pH 6.8 and then postfixed in 1% (v/v) osmium tetroxide for 2 hours at room temperature. The samples were counterstained for 1 hour with 2% uranyl acetate and embedded in low-viscosity Spurr resin. Ultrathin sections (70 nm) were cut along the longitudinal axis of the phyllids using a diamond knife on a Leica EM UC6 ultramicrotome (Leica-Reichert, Bensheim, Germany) and mounted on formvar-coated copper grids. Sections were subsequently stained with uranyl acetate and lead citrate and examined with a JEM 1200 EX II transmission electron microscope (JEOL, Tokyo, Japan) at an accelerating voltage of 80 kV.

### Image processing and analysis

Confocal images were processed using MorphoGraphX software (*39*, *74*). For volumetric analysis, 3D segmentation stacks were generated by applying the “gaussian blur” function twice before segmentation with the “ITK Watershed Auto Seeded” function with the threshold set to around 1500. The clipping plane was used to check through the samples to fix the wrongly segmented cells. Oversegmented labels were fused together manually. 3D meshes were then extracted from the volumetric stacks by “Marching Cubes 3D” function with 1-μm cube size followed by three smooth passes. “Grab label from other surface” and “Pick label in mesh1 and fill parent in mesh 2” tools were used to correlate cells with their parent labels. The clone identities were manually attributed to cells just derived from the leaf apical cell and propagated to later time points using cell lineage information. The boundary of the midrib is manually defined based on its multilayer structure.

For 2.5D (curved surface) segmentations, stacks were “gaussian blur” twice before “edge detect” with a threshold between 6000 and 13,000 followed by “edge detect angle” with a threshold between 4000 and 8000. 2.5D meshes were then created using 5-μm cube size, subdivided, and smoothed two to three times before projecting signal (4 to 6 m away from the surface). The meshes were then manually segmented.

To project the signal intensity of *PpPINApro::PpPINA-GFP-1*, surfaces were first extracted from the autofluorescence images using procedures mentioned above. The signal from *PpPINApro::PpPINA-GFP-1* at the range of 4 to 8 m was averaged and projected onto its corresponding meshes using the “Project Signal” function in MorphoGraphX software with parameter “Use absolute” set to “Yes” and “Max Signal” set to 40,000.Parent cells were manually identified and labeled between each two consecutive imaging. The cell lineage between multiple days was computed on the basis of the parent labels. The clone identities were manually labeled as in volumetric analysis.

Cellular quantification is based on cell lineage information and the topology between neighboring cells. Area expansion calculated the relative area increase from the parent cell to its daughter cells in the next time point ([Total cell area of daughter cells at Ti + 1/area of the parent cell at Ti − 1] 100%). Cell division computed the number of divisions occurring in each parent cell, which equals the number of daughter cells in the next time point minus one.

A small group of cells was manually labeled at the base of the leaf to define the leaf base region, ensuring that the selected boundary was perpendicular to the main leaf axis. This region was then used to compute distances from the base using the “Cell Distance” function in MorphoGraphX (with “Wall Weights” set to “Euclidean” to calculate absolute distance). The relative distance from the leaf base was achieved by normalizing the data with the maximum distance at the same time point. The longitudinal (Kpar) and mediolateral (Kper) growth of phyllids was computed using the PDG analysis in MorphoGraphX and projected onto the custom directions (StretchCustomX = Kpar; StretchCustomY = Kper) extracted from the heatmap of the absolute distance from the leaf base.

The new cell walls appearing in each time point were detected and highlighted by the “Select New Walls” function in MorphoGraphX. The orientation of the new wall (longitudinal or lateral) was manually identified and recorded.

To analyze the signal from *PpR2D2-1* and *PpR2D2-1-pina pinb*, confocal acquisitions were pre-processed using the ImageJ software (https://imagej.net/ij/) to select the region of interest (phyllid blades). The images were then processed using a computational pipeline described previously (*76*), which uses algorithms provided in the Python library timagetk (https://gitlab.inria.fr/mosaic/) to detect and quantify the signal from the nuclei.

### Model Description

#### Model structure

The model was implemented in the MorphoMechanX framework (*77*–*78*). The model was 2D and consisted of structures at two distinct scales: a subdivision of the phyllid into cells and a refinement of those cells into triangular finite elements; the refinement was constructed in such a way as to prevent elements from crossing cell boundaries. The cells were assigned growth parameters based on their type and position in the phyllid; these parameters were then passed down to their triangular refinement and used to determine the resultant growth of the tissue. Some cells in the model phyllid are capable of division; when a cell divided its finite elements were discarded, then the daughter cells were each triangulated into new elements. As residual stresses were released during the growth phase, this replacement caused no change in force distribution in the tissue.

#### Growth and mechanics

The finite elements are used in a growing finite element method (FEM) implementation that follows (*17*) but using 2D triangular three-node membrane elements instead of 3D six-node wedge elements. A linear St. Venant isotropic material model was used, with a Young’s modulus of 100 MPa and Poisson’s ratio of 0.3. Growth was implemented by increasing the size of the reference configuration of the elements at each growth step, with independent control of growth parallel (Kpar) or perpendicular (Kper) to a polarity direction. The rates of growth and the growth directions were specified at the cellular level, with polarity determined from the gradient of a distance field calculated from the apical cell. After each growth step, the mechanical equilibrium was found, vertex positions were updated, and any residual stresses were released.

#### Initial template

A representative mesh at 1 day was projected flat into 2D, and the cell outlines were then extracted as polygons. These polygons were used as the model template. In the model, cells are again triangulated for the FEM, and this triangulation is updated when cells divide, together with a regularization of points along the edges to maintain reasonable triangle aspect ratios for simulation. The apical cell was given a specific identity. The bottom most vertices of the template were assigned Dirichlet conditions to prevent them moving in the longitudinal direction of the phyllid.

#### Zonation and cell division

The phyllid was divided into three zones. The attachment zone at the bottom had very slow growth. Above this was a basal zone determined by distance (in cells) from the base. In this zone, cells were divided when they reached a threshold area. Division used cell polarity and picked the short pendicular to cell polarity.

The apical cell was an exception: It divided into an alternating left-right pattern at 60°. As cells grew and moved out of the basal zone, they entered the differentiation zone where they stopped dividing. The model is parameterized in such a way as to allow the growth rates to be specified as a gradient within the zones and to allow the zone sizes to change to match the phases of phyllid development and/or the influence of auxin. After each growth step, cell zones and locations were updated, and new parameters were assigned based on their positional information.

#### Midvien

At a certain time point in the simulation, a midrib was specified by choosing cells within a certain proximity of the base and the central longitudinal axis. These cells would then be assigned different growth parameters, particularly a marked reduction in growth in the medial-lateral direction. As the simulation proceeded, the midrib identity was passed on to daughter cells.

### Statistical analysis

Data were extracted and analyzed using the Python 3.9 matplotlib library (https://matplotlib.org/stable/). Plots were generated using Excel, Python 3.9, Matplotlib, and Seaborn. In box plots, the central line represents the median, red dots indicate the mean, boxes span the interquartile range (first to third), and whiskers represent the 5th to 95th percentiles. The violin plots display the kernel probability distribution of the 5th to 95th percentiles of the data. Statistical significance was assessed with a two-sided Mann-Whitney U test. To analyze growth patterns relative to the base of the leaf, the leaf was divided into 10 equal-length bins along its longitudinal axis. Median growth values within each bin were computed and visualized using a smooth spline interpolation, implemented via the make_interp_spline function from the scipy.interpolate module of the SciPy library. The variability in growth was represented by shaded regions around the spline curve, corresponding to the interquartile range (first and third quartiles) along the *x* axis.

## Supplementary Materials

### The PDF file includes:

Figs. S1 to S15 Legends for movies S1 to S15 Legend for table S1 References
