# Supplementary material for: Morphogenesis of moss leaf-like organs through variations in deeply shared developmental principles
Source: Sci Adv. 2026 Apr 15;12(16):eaee6959. doi: 10.1126/sciadv.aee6959 (PMC13082339; doi:10.1126/sciadv.aee6959)
Supplement: Supplementary file 1 — Figs. S1 to S15 Legends for movies S1 to S15 Legend for table S1 References [file sciadv.aee6959_sm.pdf]

Science Advances
AAAS

Supplementary Materials for

Morphogenesis of moss leaf-like organs through variations in deeply shared developmental principles

Wenye Lin *et al.*

Corresponding author: Richard S. Smith, richard.smith@jic.ac.uk; Yoan Coudert, yoan.coudert@cnrs.fr; Daniel Kierzkowski, daniel.kierzkowski@umontreal.ca

Sci. Adv. **12**, eaee6959 (2026)
DOI: 10.1126/sciadv.aee6959

The PDF file includes:

Figs. S1 to S15
Legends for movies S1 to S15
Legend for table S1
References

Other Supplementary Material for this manuscript includes the following:

Movies S1 to S15
Table S1

SUPPLEMENTARY FIGURES

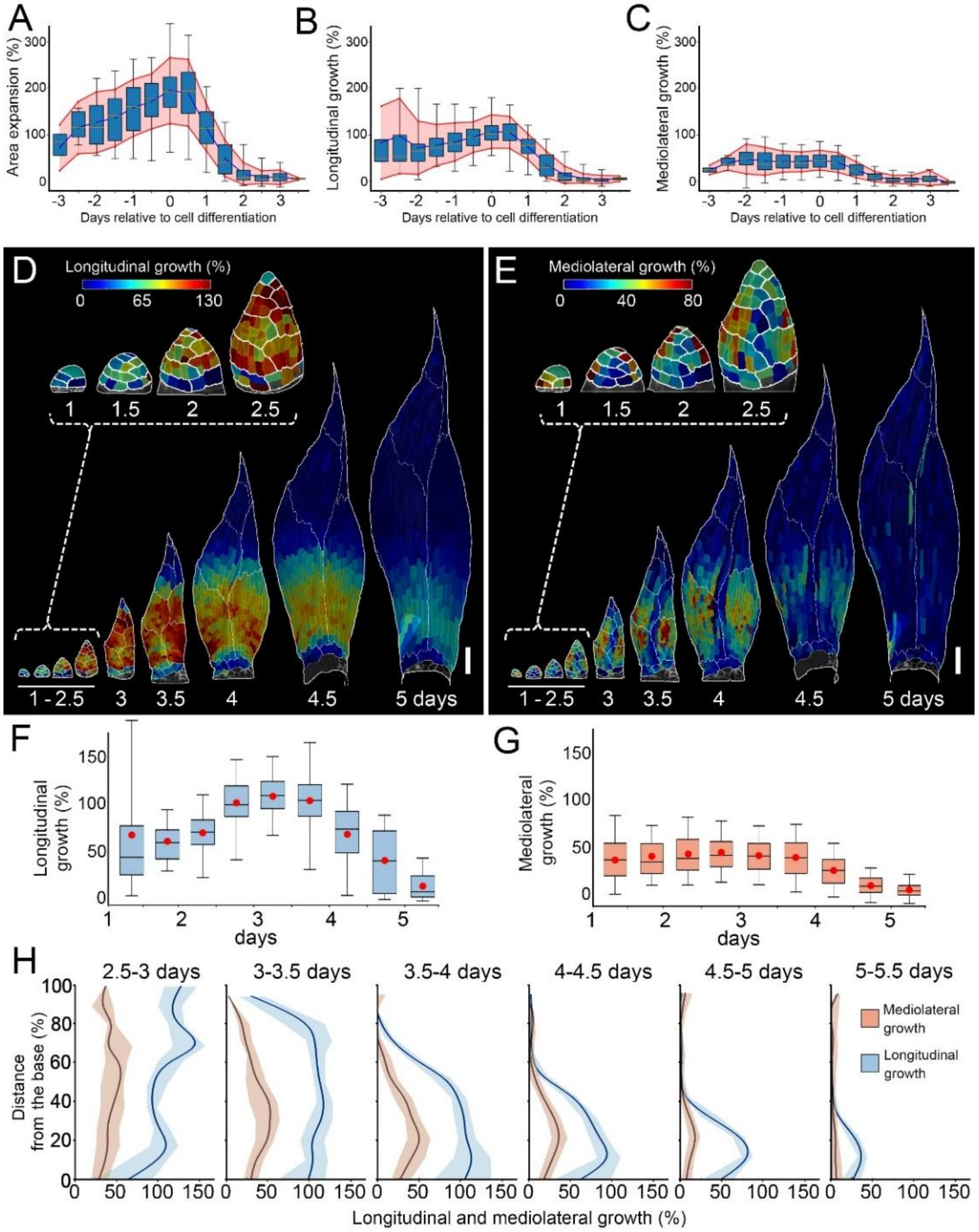

Fig. S1. Mediolateral and longitudinal growth in the phyllid are independent from cell-lineage. (A-C) Quantification of cell area expansion (A), cellular growth along longitudinal (B), and mediolateral (C) axis of the upper phyllid of *Physcomitrium patens* in days relative to cell differentiation (last division). **(D-E)** Heat-maps of cellular growth along longitudinal (D) and mediolateral (E) organ axis. Heat values are displayed at the earlier time point. **(F-G)** Quantifications of cellular growth along longitudinal (F), mediolateral (G) in the upper phyllid. Boxes contain the second and third quartile and whiskers 90 % of data. Lines represent the median and the red dots the mean (n=18, 46, 91, 226, 490, 799, 931, 939 and 938 cells at consecutive time points; three independent time-lapse series). **(H)** Quantification of cellular growth along longitudinal and mediolateral organ axis as a function of the normalized distance from its base. Shades contain the second and third quartile and lines indicate the median. Scale bars = 100 µm. Related to Fig. 1.

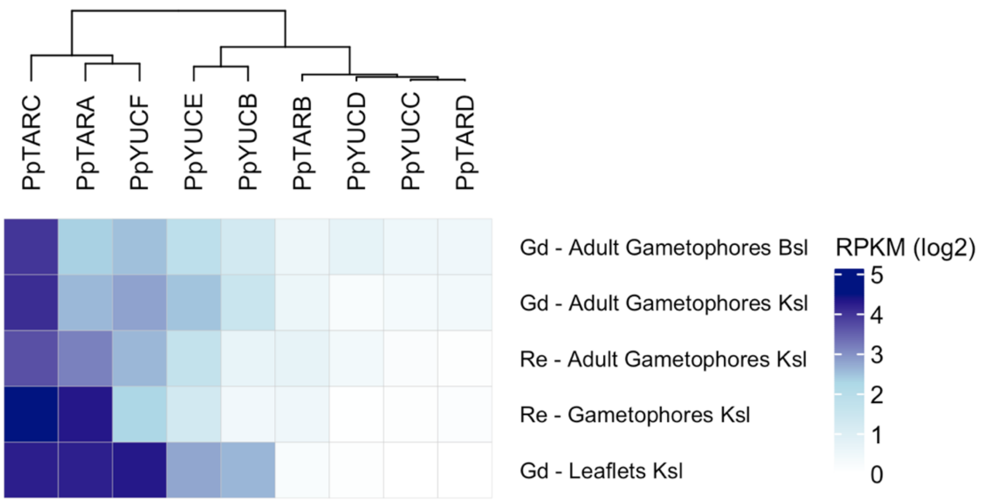

Fig. S2. Expression of genes involved in auxin synthesis. Heatmap showing expression levels of *PpTAR* and *PpYUC* genes in gametophores and phyllids from Gransden (Gd) and Reute (Re) ecotypes grown on KNOP solid medium (Ksl) or BCD solid medium (Bsl). All samples were normalized to RPKM.

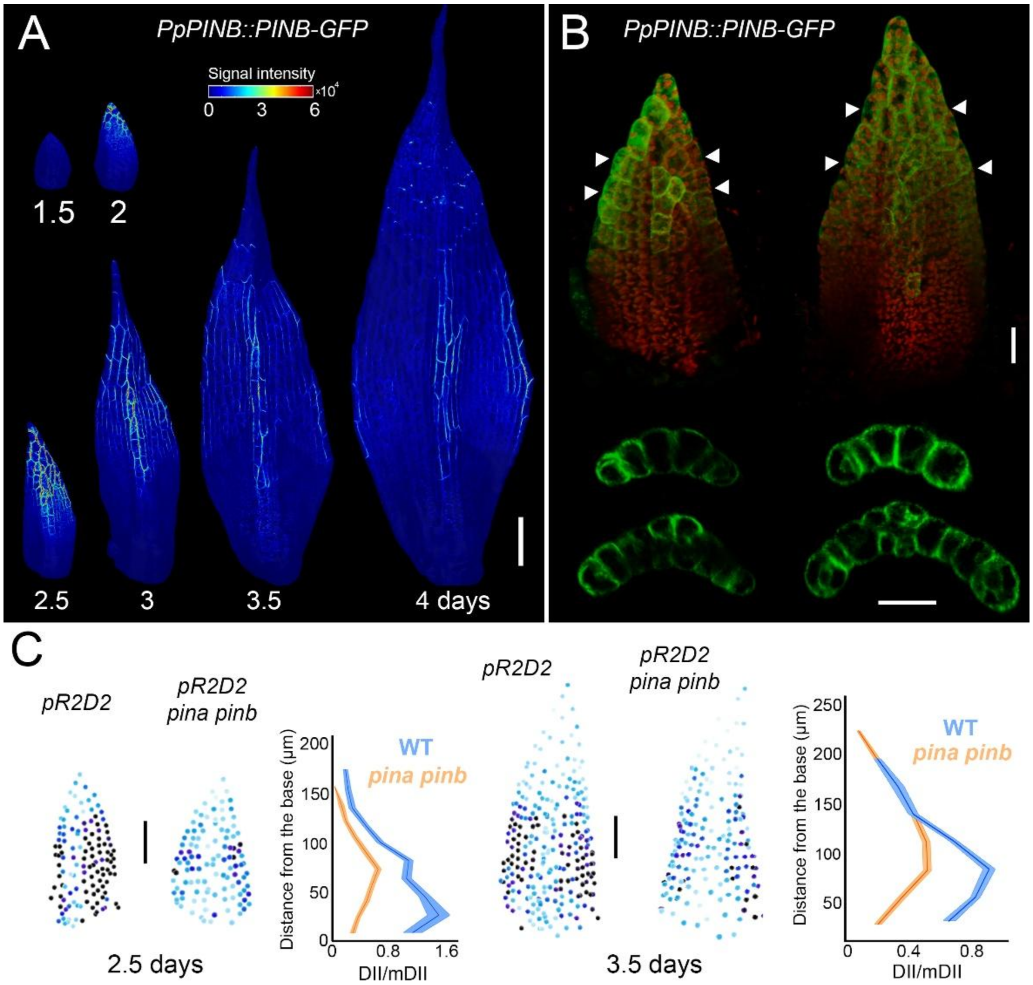

Fig. S3. The localization of PINB auxin efflux carrier and the quantification of R2D2 signal distribution. (A) Expression of *PpPINB::PINB-GFP* in the consecutive stages of upper phyllid development. Heatmap represents the intensity of PINB-GFP signal. **(B)** Maximal projections (top) and cross-sections (bottom) of confocal stacks with PINB-GFP signal in green and chloroplast autofluorescence in red. Arrowheads indicate the positions of the cross-sections**. (C)** Images of segmented nuclei in phyllids at 2.5 and 3.5 days. Each nucleus is color-coded according to the relative level of auxin sensing (DII/mDII signal ratio) (left) and quantification of DII/mDII signal ratio as a function of the absolute distance from the phyllid base. The solid line represents the mean ratio within each spatial bin, and the shaded area shows the corresponding Standard Error of the Mean SEM for the pooled data.. Scale bar, 100 µm (A) and 20 µm (B). Related to Fig. 2.

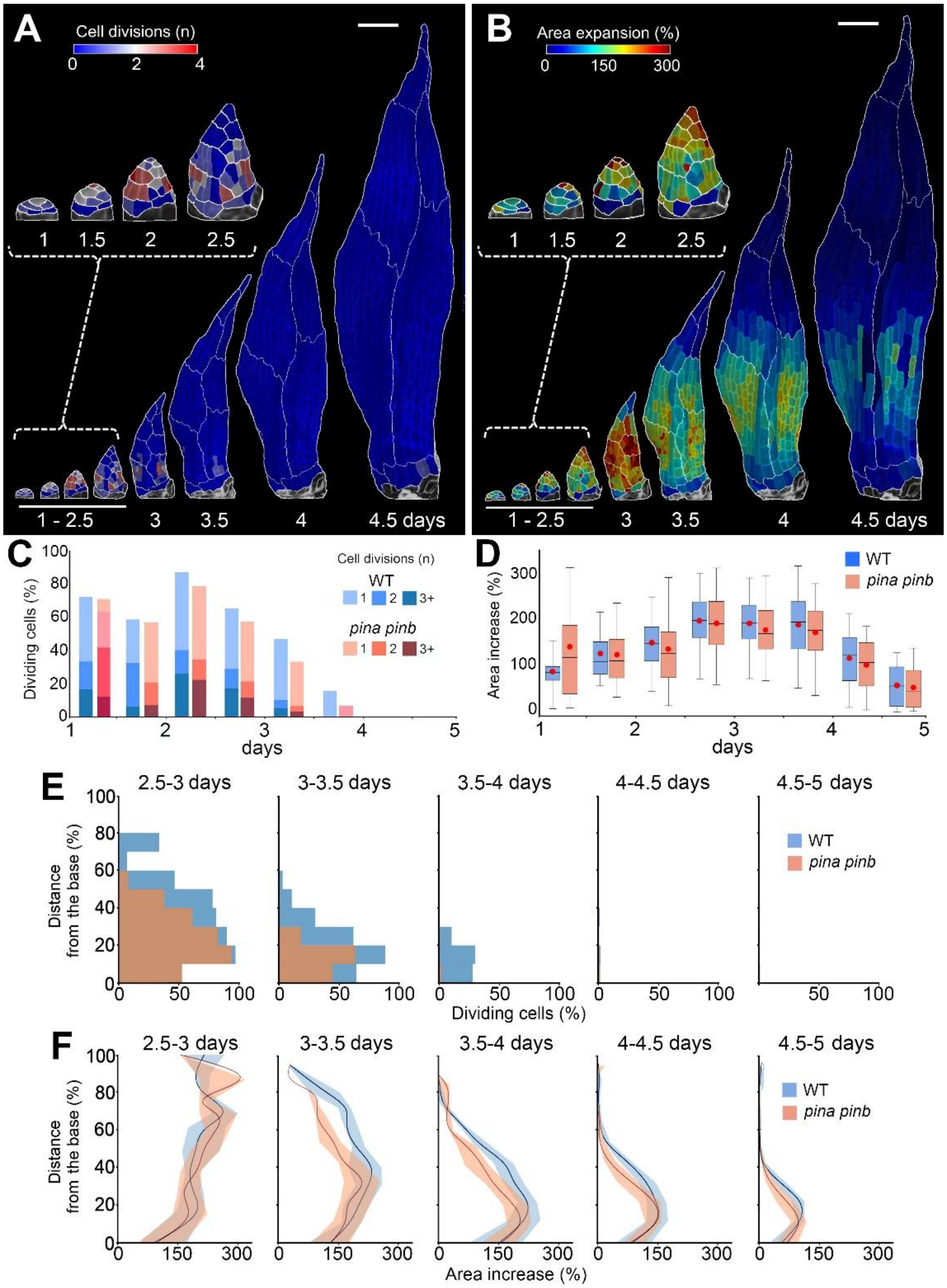

Fig. S4. Removing PINA and PINB activity reduces cell division of phyllids. (A-B) Heat-maps of cell divisions (A) and area increase (B) for the upper phyllid of *pina pinb* mutant. Heat values are displayed on the earlier time point. **(C-D)** Quantifications of cell divisions (C), and area increase (D) in the upper phyllid of the *pina pinb* mutant. Boxes contain the second and third quartile and whiskers 90 % of data (n=17, 37, 82, 195, 362, 510, 558 and 567 cells at consecutive time points; three time-lapse series). Lines represent the median and red dots the mean. **(E-F)** Quantification of the number of dividing cells (E) and area increases (F) as a function of the normalized distance from the organ base. Shades contain the second and third quartile and lines indicate the median. Scale bars = 100 µm. Related to Fig. 2.

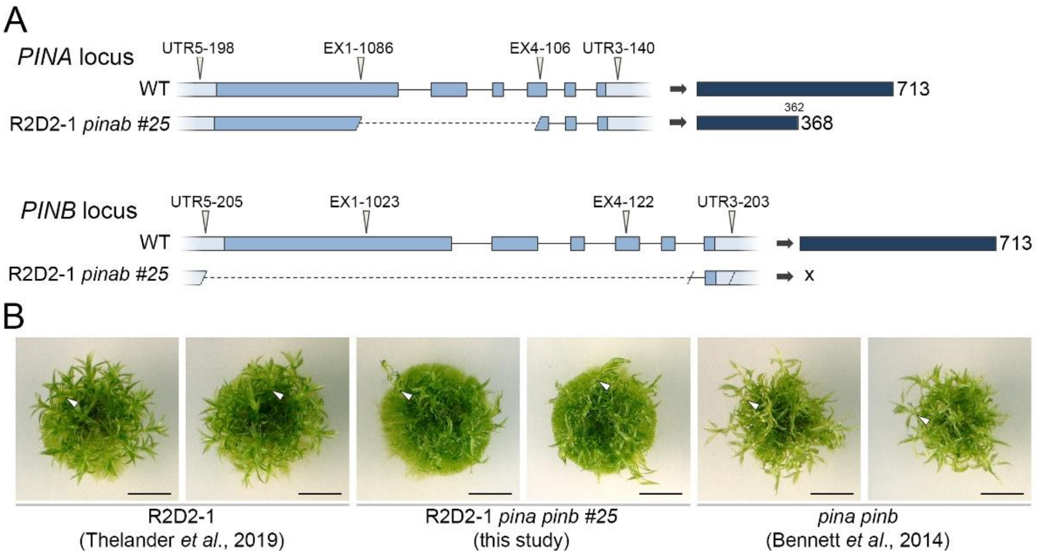

Fig. S5. Genetic characterization and phenotyping of the R2D2-1 pinab line. (A) Schematic representation of the WT *PINA* and *PINB* genomic locus showing the positions of four specific single guide RNAs (arrowheads) and the resulting genomic edits identified by sequencing in the R2D2-1 *pinab* #25 line (left). Blue boxes indicate exons; light blue boxes indicate 5′and 3′ untranslated regions (UTRs); introns are shown as solid black lines; dashed black lines indicate deleted regions of the genes. Predicted protein products resulting from an *in silico* translation of the edited genomic sequences (right). WT PINA and PINB proteins are 713 amino acids (AA) long. The mutant PINA protein is truncated to 368 AA, with a frameshift starting at position 362. **(B)** Photographs of representative 4-week-old clones of the R2D2-1 *pina pinb* #25 line generated in this study, compared to the original R2D2-1 genetic background. Gametophores (indicated by white arrowheads) in the R2D2-1 *pina pinb* #25 line display developmental alterations similar to those reported for the *pina pinb* double mutant in Bennett et al. (2014), confirming the efficiency of gene disruption. Note that the alterations of protonemal development observed in the R2D2-1 *pina pinb* #25 line were also present in the original R2D2-1 line and are therefore independent of PIN gene knockout. Scale bars, 0.5 cm. Related to Fig. 2.

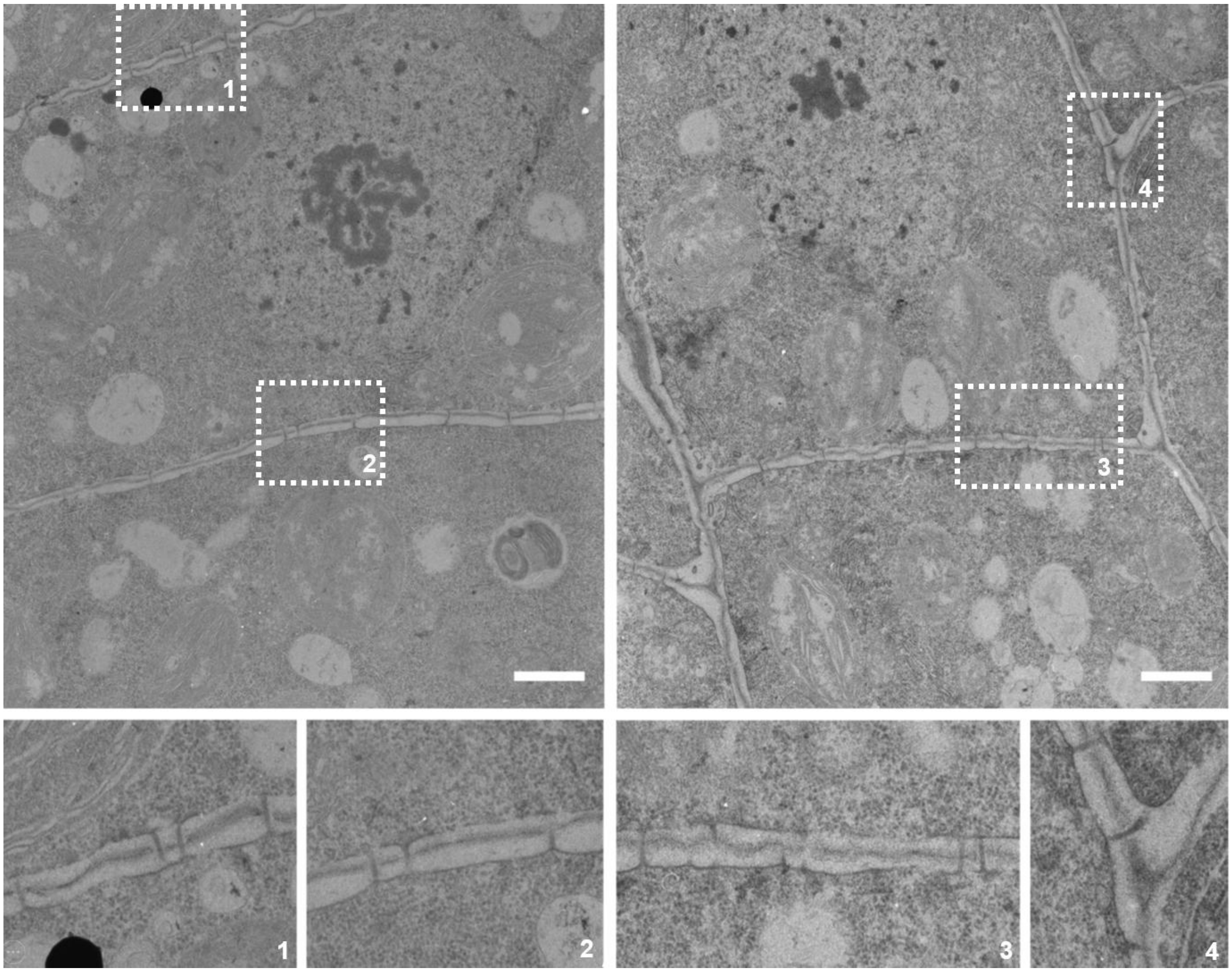

Fig. S6. Visualization of the plasmodesmata in the anticlinal wall of the developing phyllids. Representative transmission electron microscopy micrographs of phyllids at around 2.5-3.5 days after initiation. Insets indicate close up view of the plasmodesmata. Scale bars, 1 µm. Related to Fig. 2.

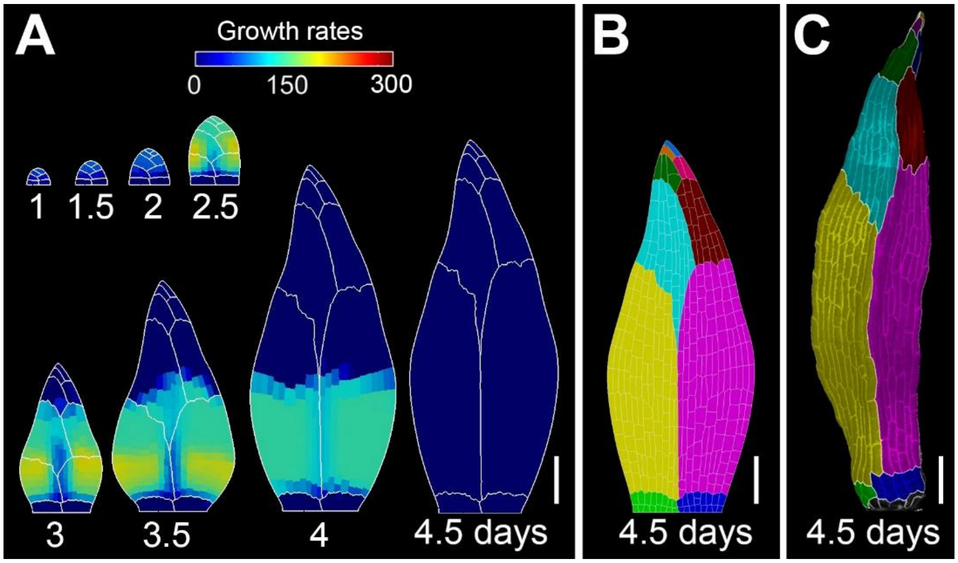

Fig. S7. Model of phyllid growth with reduced cell division zone and the timing of cell proliferation. (A) Model output colored by areal specified growth rate. **(B)** Resultant shape from the model colored by areal specified growth rate. **(C)** Fully developed upper phyllid of *pina pinb* mutant with colors marking sectors originating from the apical cell. Note that modelled phyllid is much shorter than double mutant. Scale bars, 100 µm. Related to Fig. 2.

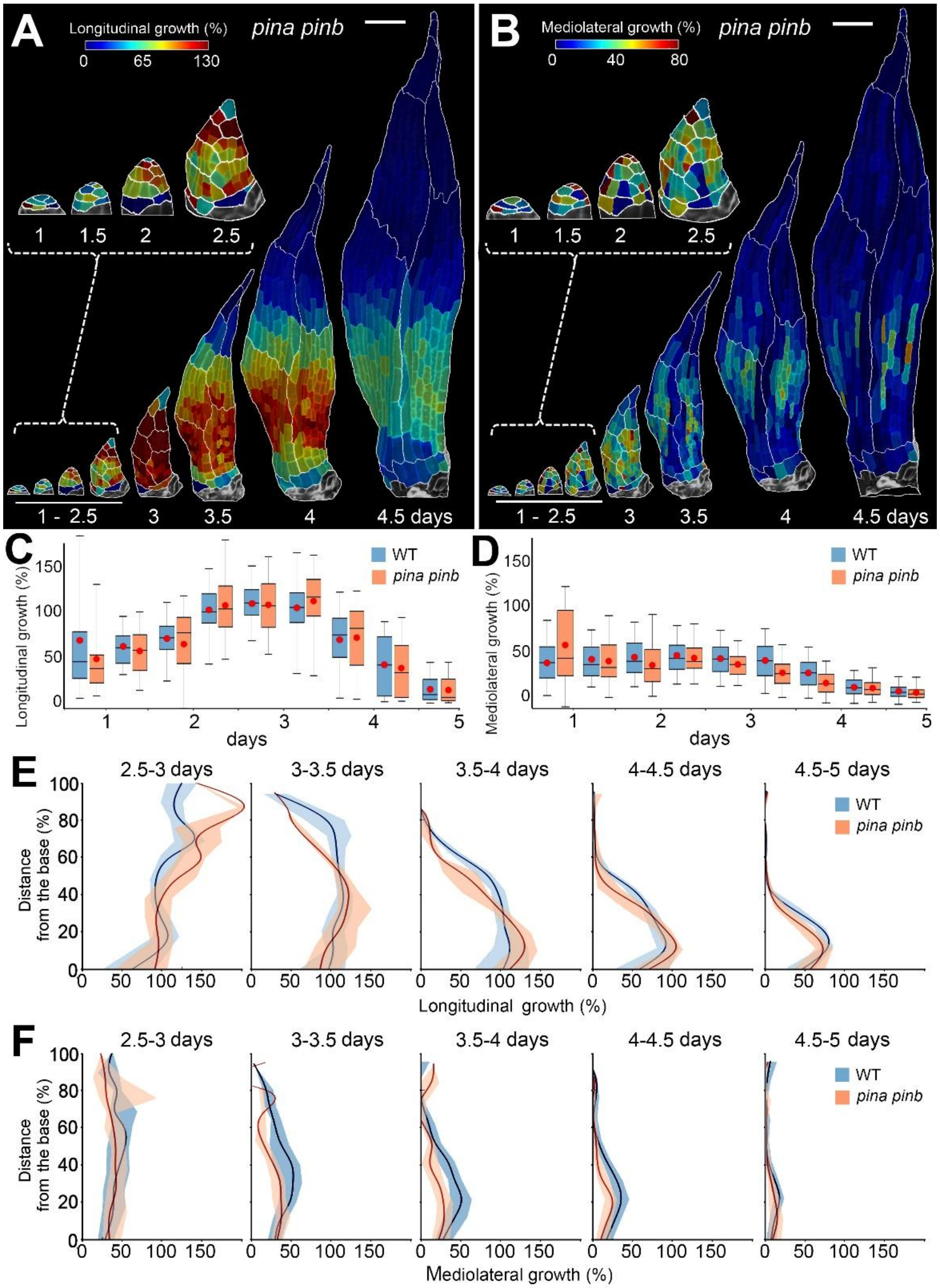

Fig. S8. Removing PINA and PINB activity increases longitudinal growth and decreases mediolateral growth. **(A-B)** Heat-maps of cellular growth along longitudinal (A) and mediolateral (B) axis of the upper phyllid of the *pina pinb* mutant. Heat values are displayed on the earlier time point. **(C-D)** Quantifications of cellular growth along longitudinal (C), medio-lateral (D) in the upper phyllid of the *pina pinb* mutant. Boxes contain the second and third quartile and whiskers 90 % of data (n=17, 37, 82, 195, 362, 510, 558 and 567 cells at consecutive time points; three time-lapse series). Lines represent the median and the red dots the mean. **(E-F)** Quantification of cellular growth along longitudinal (E), medio-lateral (F) as a function of the normalized distance from the organ base. Shades contain the second and third quartile and lines indicate the median. Scale bars = 100 µm. Related to Fig. 2.

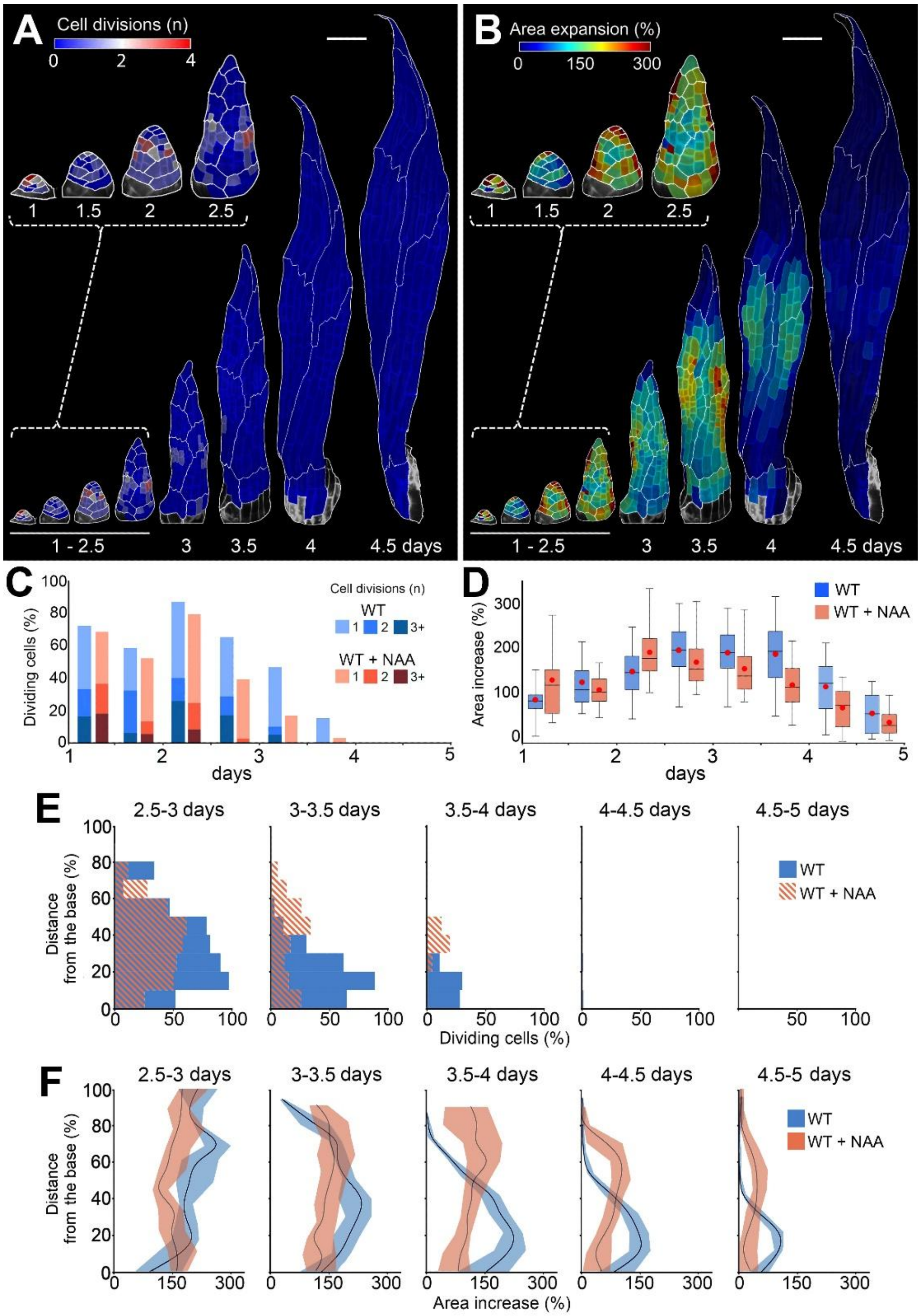

Fig. S9. Auxin treatment perturbs basipetal gradients of cell proliferation and growth in the upper phyllid. (A-B) Heat-maps of cell divisions (A) and area increase (B) for the upper phyllid upon NAA treatment. Heat values are displayed on the earlier time point. **(C-D)** Quantifications of cell divisions (C), and area increases (D) in the upper phyllid upon NAA treatment. Boxes contain the second and third quartile and whiskers 90 % of data (n=22, 52, 93, 193, 271, 321, 325 and 320 cells at consecutive time points; three time-lapse series). Lines represent the median and red dots the mean. **(E-F)** Quantification of the number of dividing cells (E) and area increases (F) as a function of the distance from the organ base. Shades contain the second and third quartile and lines indicate the median. Scale bars = 100 µm. Related to Fig. 3.

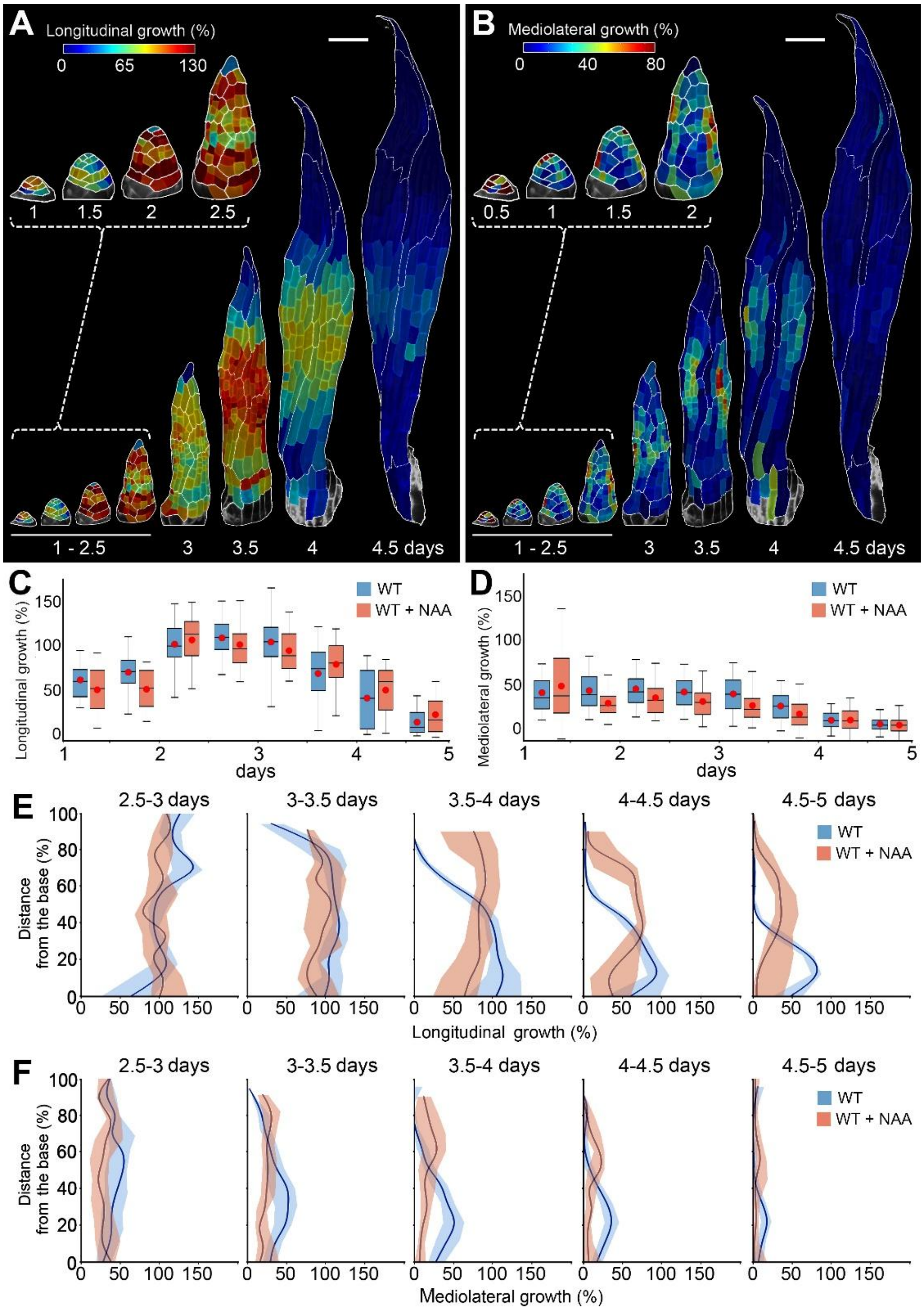

Fig. S10. Auxin treatment perturbs basipetal gradients of growth in the upper phyllid. (A-B) Heat-maps of cellular growth along longitudinal (A) and mediolateral (B) axis of the upper phyllid upon auxin treatment. Heat values are displayed on the earlier time point. **(C-D)** Quantifications of cellular growth along longitudinal (C), medio-lateral (D) in the upper phyllid upon auxin treatment. Boxes contain the second and third quartile and whiskers 90 % of data (n=22, 52, 93, 193, 271, 321, 325 and 320 cells at consecutive time points; three time-lapse series). Lines represent the median and the red dots the mean. **(E-F)** Quantification of cellular growth along longitudinal (E), medio-lateral (F) as a function of the normalized distance from the organ base. Shades contain the second and third quartile and lines indicate the median. Scale bars = 100 μm. Related to Fig. 3.

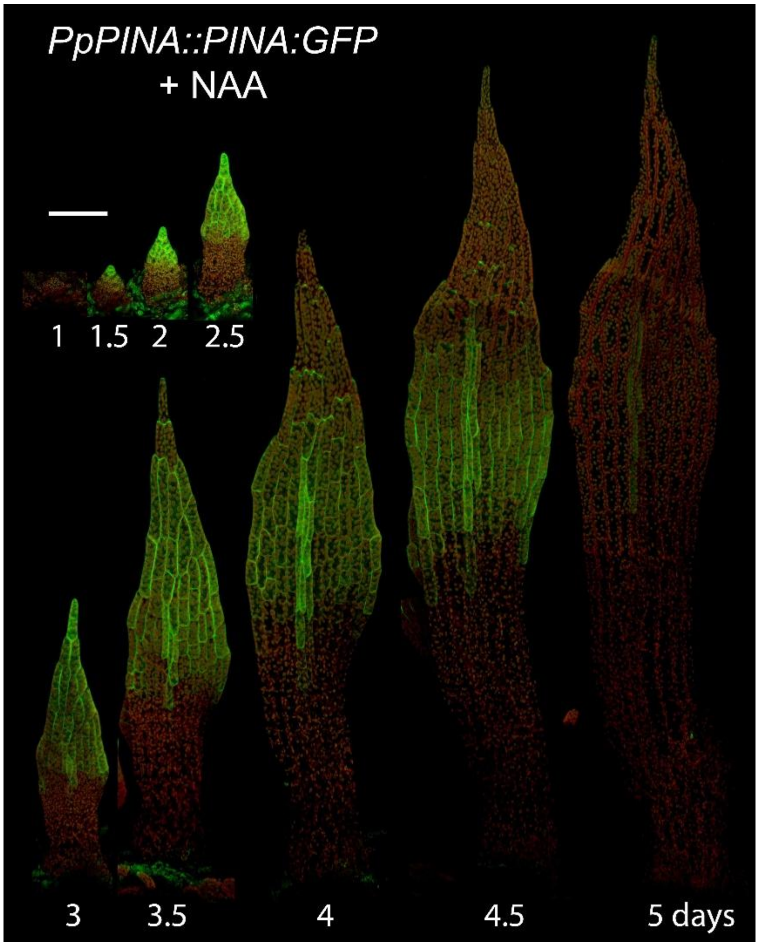

Fig. S11. Auxin treatment does not affect the localization of PINA auxin efflux carrier. Time-lapse imaging of the localization patterns of *PpPINA::PINA-GFP* during growth of the upper phyllis in the wild-type treated with NAA. PINA-GFP in green, autofluorescence in red. Scale bar, 100 µm. Related to Fig. 3.

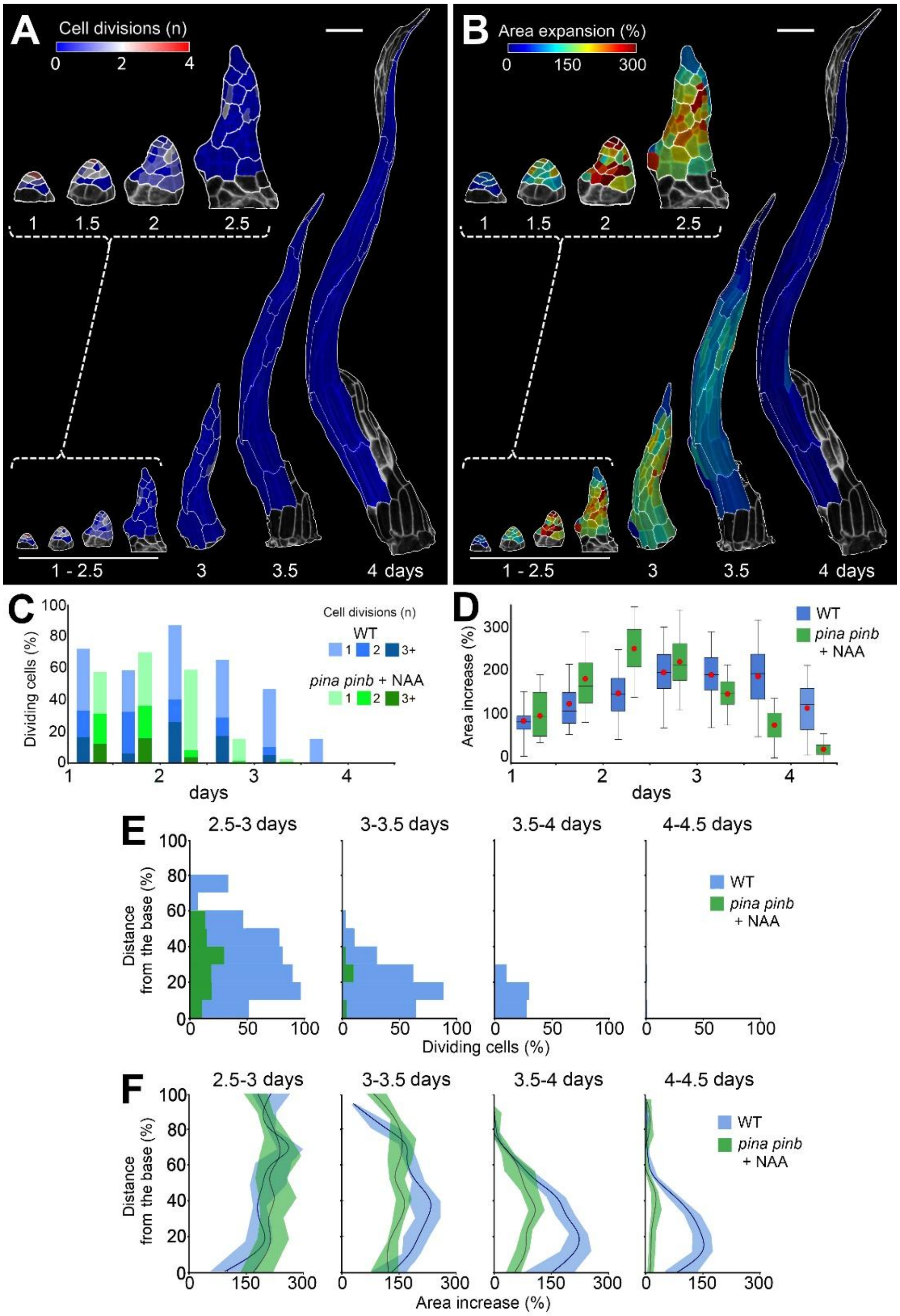

Fig. S12. Auxin treatment of the *pina pinb* phyllid causes early cessation of cell divisions and growth. (A-B) Heat-maps of cell divisions (A) and area increase (B) for the upper phyllid of *pina pinb* upon NAA treatment. Heat values are displayed on the earlier time point. **(C-D)** Quantifications of cell divisions (C), and area increases (D) in the upper phyllid in *pina pinb* mutant upon NAA treatment. Boxes contain the second and third quartile and whiskers 90 % of data (n=25, 52, 119, 201, 234, 239 and 237 cells at consecutive time points; three time-lapse series). Lines represent the median and red dots the mean **(E-F)** Quantification of the number of dividing cells (E) and area increases (F) as a function of the normalized distance from the organ base. Shades contain the second and third quartile and lines indicate the median. Scale bars = 100 µm. Related to Fig. 3.

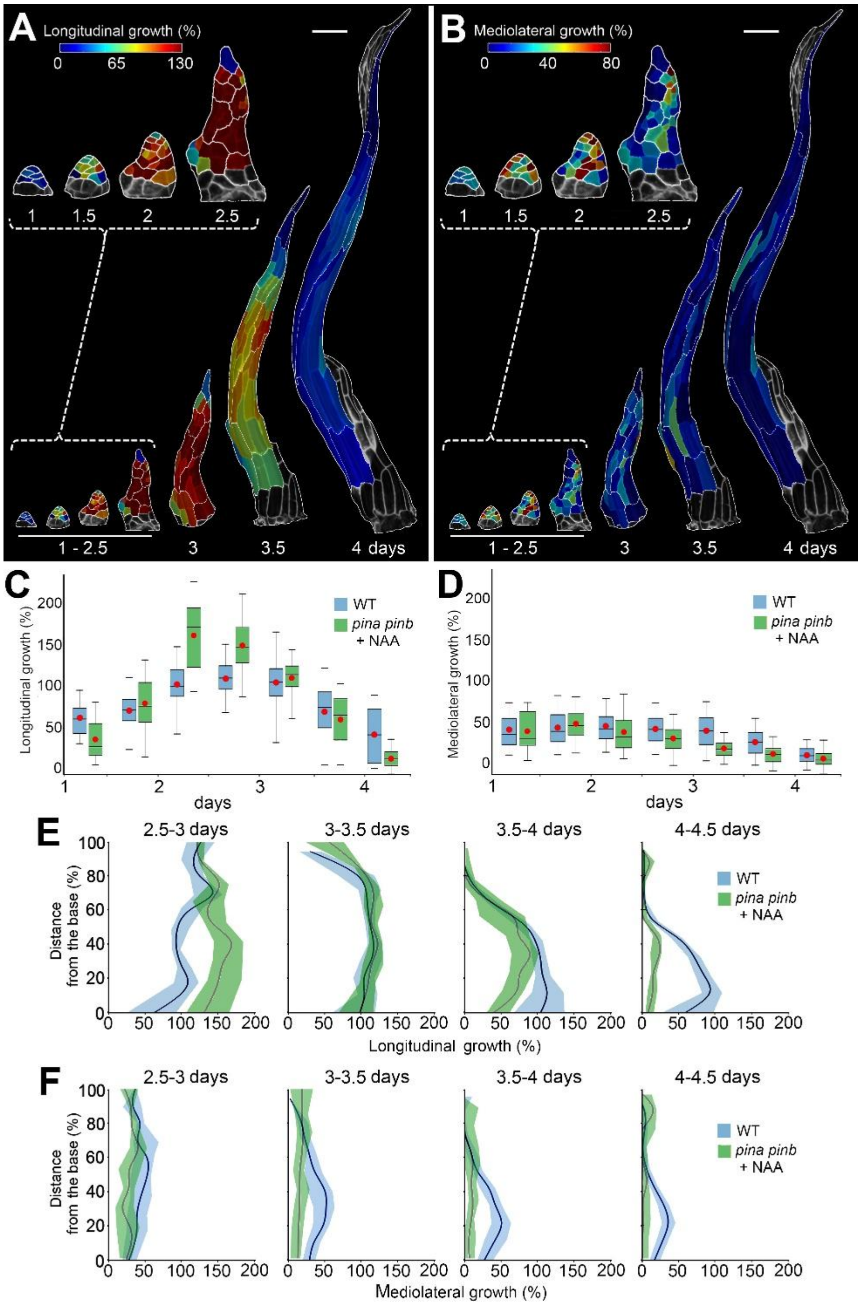

Fig. S13. Auxin treatment of the *pina pinb* phyllid strongly increases longitudinal growth while blocking growth along the mediolateral axis. (A-B) Heat-maps of cellular growth along longitudinal (A) and mediolateral (B) axis in the upper phyllid of the *pina pinb* mutant upon auxin treatment. Heat values are displayed at the earlier time point. **(C-D)** Quantifications of cellular growth along longitudinal (C), medio-lateral (D) in the upper phyllid of the *pina pinb* mutant upon auxin treatment. Boxes contain the second and third quartile and whiskers 90 % of data (n=25, 52, 119, 201, 234, 239 and 237 cells at consecutive time points; three time-lapse series). Lines represent the median and the red dots the mean. **(E-F)** Quantification of cellular growth along longitudinal (E), medio-lateral (F) as a function of the normalized distance from the organ base. Shades contain the second and third quartile and lines indicate the median. Scale bars = 100 µm. Related to Fig. 3.

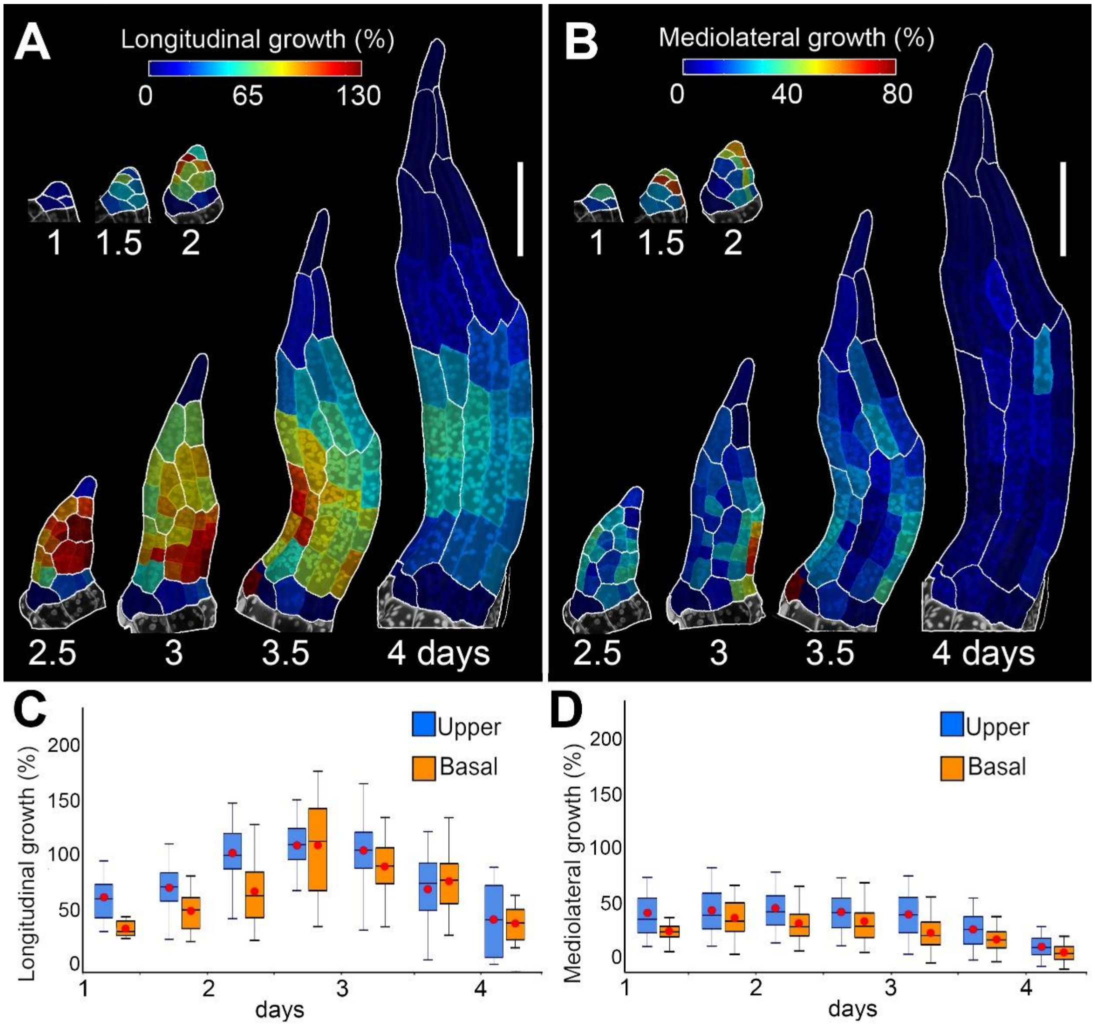

Fig. S14. Growth of the basal phyllid is mainly longitudinal. (A-B) Heat-maps of cellular growth along longitudinal (A) and mediolateral (B) axis in the basal phyllid. Heat values are displayed at the earlier point. **(C-D)** Quantifications of cellular growth along longitudinal (C), medio-lateral (D) in the basal phyllid. Boxes contain the second and third quartile and whiskers 90 % of data (n=15, 43, 94, 158, 238, 201 and 205 cells at consecutive time points; three time-lapse series). Lines represent the median and the red dots the mean. Scale bars = 100 µm. Related to Fig. 4.

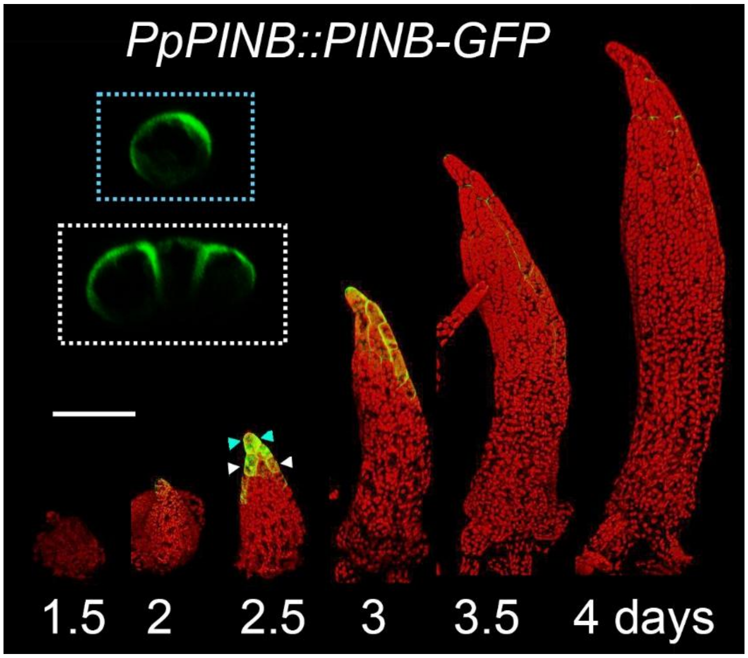

Fig. S15. The localization of PINB auxin efflux carrier. Expression of *PpPINB::PINB-GFP* in the consecutive stages of basal phyllid development. Maximal projections of confocal stacks with PINB-GFP signal in green and chloroplast autofluorescence in red. Insets: close-up view of the phyllid cross-sections. Arrowheads indicate the position of the cross sections shown. Scale bar, 100 μm. Related to Fig. 4.

CAPTIONS FOR THE SUPPLEMENTARY MOVIES

Movie S1. Cell division and area expansion in the wild-type upper phyllid of *Physcomitrium patens*.

Movie S2. Longitudinal growth and mediolateral growth in the wild-type upper phyllid of *Physcomitrium patens*.

Movie S3. Model of wild-type upper phyllid of *Physcomitrium patens*.

Movie S4: Cell division and area expansion in the *pina pinb* upper phyllid.

Movie S5: Model of *pina pinb* upper phyllid with reduced cell divisions compared to WT.

Movie S6: Longitudinal growth and mediolateral growth in *pina pinb* upper phyllid.

Movie S7: Model of *pina pinb* upper phyllid with reduced cell divisions and increased growth anisotropy as compared to WT.

Movie S8: Model of wild-type upper phyllid with cell divisions eliminated after Phase #1 and an increased growth anisotropy.

Movie S9: Cell division and area expansion in the wild-type upper phyllid treated with NAA.

Movie S10: Longitudinal growth and mediolateral growth in the wild-type upper phyllid treated with NAA.

Movie S11: Cell division and area expansion in the *pina pinb* upper phyllid treated with NAA.

Movie S12: Longitudinal growth and mediolateral growth in *pina pinb* upper phyllid treated with NAA.

Movie S13: Cell division and area expansion in the wild-type basal phyllid

Movie S14: Longitudinal growth and mediolateral growth in the wild-type basal phyllid.

Movie S15: Model of wild-type basal phyllid.

https://doi.org/10.5061/dryad.kkwh70sjp

Data.zip: File containing all the numerical data needed to reproduce the results.

Models.zip: All models and starting templets for the models.

MDX-2.0.3-117-Ubuntu24.04-Cuda12.8.deb: Copy of MorphoDynamX software with Cuda.

MDX-2.0.3-117-Ubuntu24.04.deb: Copy of MorphoDynamX software without Cuda.

Table S1: Parameters of the models for phyllid development.

REFERENCES

1. A. M. F. Tomescu, Megaphylls, microphylls and the evolution of leaf development. *Trends Plant Sci.* **14**, 5–12 (2009).

2. H. Nelissen, N. Gonzalez, D. Inzé, Leaf growth in dicots and monocots: So different yet so alike. *Curr. Opin. Plant Biol.* **33**, 72–76 (2016).

3. S. K. Floyd, J. L. Bowman, Distinct developmental mechanisms reflect the independent origins of leaves in vascular plants. *Curr. Biol.* **16**, 1911–1917 (2006).

4. C. J. Harrison, S. B. Corley, E. C. Moylan, D. L. Alexander, R. W. Scotland, J. A. Langdale, Independent recruitment of a conserved developmental mechanism during leaf evolution. *Nature* **434**, 509–514 (2005).

5. W. Lin, Y. Wang, Y. Coudert, D. Kierzkowski, Leaf morphogenesis: Insights from the moss *Physcomitrium patens*. *Front. Plant Sci.* **12**, 736212 (2021).

6. C. J. Harrison, A. H. K. Roeder, E. M. Meyerowitz, J. A. Langdale, Local cues and asymmetric cell divisions underpin body plan transitions in the moss *Physcomitrella patens*. *Curr. Biol.* **19**, 461–471 (2009).

7. A. Maugarny-Calès, P. Laufs, Getting leaves into shape: A molecular, cellular, environmental and evolutionary view. *Development* **145**, dev161646 (2018).

8. D. Wilson-Sánchez, N. Bhatia, A. Runions, M. Tsiantis, From genes to shape in leaf development and evolution. *Curr. Biol.* **32**, R1215–R1222 (2022).

9. C. D. Whitewoods, B. Gonçalves, J. Cheng, M. Cui, R. Kennaway, K. Lee, C. Bushell, M. Yu, C. Piao, E. Coen, Evolution of carnivorous traps from planar leaves through simple shifts in gene expression. *Science* **367**, 91–96 (2020).

10. A. Runions, M. Tsiantis, P. Prusinkiewicz, A common developmental program can produce diverse leaf shapes. *New Phytol.* **216**, 401–418 (2017).

11. K. R. Challa, M. Rath, A. N. Sharma, A. K. Bajpai, S. Davuluri, K. K. Acharya, U. Nath, Active suppression of leaflet emergence as a mechanism of simple leaf development. *Nat. Plants* **7**, 1264–1275 (2021).

12. D. Kierzkowski, A. Runions, F. Vuolo, S. Strauss, R. Lymbouridou, A.-L. Routier-Kierzkowska, D. Wilson-Sánchez, H. Jenke, C. Galinha, G. Mosca, Z. Zhang, C. Canales, R. Dello Ioio, P. Huijser, R. S. Smith, M. Tsiantis, A growth-based framework for leaf shape development and diversity. *Cell* **177**, 1405–1418.e17 (2019).

13. P. M. Donnelly, D. Bonetta, H. Tsukaya, R. E. Dengler, N. G. Dengler, Cell cycling and cell enlargement in developing leaves of Arabidopsis. *Dev. Biol.* **215**, 407–419 (1999).

14. M. Andriankaja, S. Dhondt, S. De Bodt, H. Vanhaeren, F. Coppens, L. De Milde, P. Mühlenbock, A. Skirycz, N. Gonzalez, G. T. S. Beemster, D. Inzé, Exit from proliferation during leaf development in *Arabidopsis thaliana*: A not-so-gradual process. *Dev. Cell* **22**, 64–78 (2012).

15. M. Das Gupta, U. Nath, Divergence in patterns of leaf growth polarity is associated with the expression divergence of miR396. *Plant Cell* **27**, 2785–2799 (2015).

16. S. Fox, P. Southam, F. Pantin, R. Kennaway, S. Robinson, G. Castorina, Y. E. Sánchez-Corrales, R. Sablowski, J. Chan, V. Grieneisen, A. F. M. Marée, J. A. Bangham, E. Coen, Spatiotemporal coordination of cell division and growth during organ morphogenesis. *PLoS Biol.* **16**, e2005952 (2018).

17. E. E. Kuchen, S. Fox, P. B. De Reuille, R. Kennaway, S. Bensmihen, J. Avondo, G. M. Calder, P. Southam, A. Bangham, E. Coen, Generation of leaf shape through early patterns of growth and tissue polarity. *Science* **335**, 1092–1096 (2012).

18. X.-M. Li, H. Jenke, S. Strauss, C. Bazakos, G. Mosca, R. Lymbouridou, D. Kierzkowski, U. Neumann, P. Naik, P. Huijser, S. Laurent, R. S. Smith, A. Runions, M. Tsiantis, Cell-cycle-linked growth reprogramming encodes developmental time into leaf morphogenesis. *Curr. Biol.* **34**, 541–556.e15 (2024).

19. C. Le Gloanec, A. Gómez-Felipe, V. Alimchandani, E. Branchini, A. Bauer, A.-L. Routier-Kierzkowska, D. Kierzkowski, Modulation of cell differentiation and growth underlies the shift from bud protection to light capture in cauline leaves. *Plant Physiol.* **196**, 1214–1230 (2024).

20. N. Shankar, P. Sunkara, U. Nath, A double-negative feedback loop between miR319c and JAW-TCPs establishes growth pattern in incipient leaf primordia in *Arabidopsis thaliana*. *PLOS Genet.* **19**, 1010978 (2023).

21. Z. Zhang, A. Runions, R. A. Mentink, D. Kierzkowski, M. Karady, B. Hashemi, P. Huijser, S. Strauss, X. Gan, K. Ljung, M. Tsiantis, A WOX/auxin biosynthesis module controls growth to shape leaf form. *Curr. Biol.* **24**, 4857–4868 (2020).

22. S. Vanneste, J. Friml, Auxin: A trigger for change in plant development. *Cell* **136**, 1005–1016 (2009).

23. D. Kierzkowski, M. Lenhard, R. Smith, C. Kuhlemeier, Interaction between meristem tissue layers controls phyllotaxis. *Dev. Cell* **26**, 616–628 (2013).

24. D. Reinhardt, E.-R. Pesce, P. Stieger, T. Mandel, K. Baltensperger, M. Bennett, J. Traas, J. Friml, C. Kuhlemeier, Regulation of phyllotaxis by polar auxin transport. *Nature* **426**, 255–260 (2003).

25. M. G. Heisler, C. Ohno, P. Das, P. Sieber, G. V. Reddy, J. A. Long, E. M. Meyerowitz, Patterns of auxin transport and gene expression during primordium development revealed by live imaging of the arabidopsis inflorescence meristem. *Curr. Biol.* **15**, 1899–1911 (2005).

26. A. E. Richardson, J. Cheng, R. Johnston, R. Kennaway, B. R. Conlon, A. B. Rebocho, H. Kong, M. J. Scanlon, S. Hake, E. Coen, Evolution of the grass leaf by primordium extension and petiole-lamina remodeling. *Science* **374**, 1377–1381 (2021).

27. K. Abley, P. B. De Reuille, D. Strutt, A. Bangham, P. Prusinkiewicz, A. F. M. Marée, V. A. Grieneisen, E. Coen, An intracellular partitioning-based framework for tissue cell polarity in plants and animals. *Development* **140**, 2061–2074 (2013).

28. G. D. Bilsborough, A. Runions, M. Barkoulas, H. W. Jenkins, A. Hasson, C. Galinha, P. Laufs, A. Hay, P. Prusinkiewicz, M. Tsiantis, Model for the regulation of *Arabidopsis thaliana* leaf margin development. *Proc. Natl. Acad. Sci. U.S.A.* **108**, 3424–3429 (2011).

29. C. Gao, X. Liu, N. De Storme, K. H. Jensen, Q. Xu, J. Yang, X. Liu, S. Chen, H. Juel Martens, A. Schulz, J. Liesche, Directionality of plasmodesmata-mediated transport in arabidopsis leaves supports auxin channeling. *Curr. Biol.* **30**, 1970–1977.e4 (2020).

30. C. S. Bascom, S. Z. Wu, K. Nelson, J. Oakey, M. Bezanilla, Long-term growth of moss in microfluidic devices enables subcellular studies in development. *Plant Physiol.* **172**, 28–37 (2016).

31. E. I. Barker, N. W. Ashton, Heteroblasty in the moss, *Aphanoregma patens* (*Physcomitrella patens*), results from progressive modulation of a single fundamental leaf developmental programme. *J. Bryol.* **35**, 185–196 (2013).

32. R. J. Dennis, C. D. Whitewoods, C. J. Harrison, Quantitative methods in like-for-like comparative analyses of *Aphanorrhegma* (Physcomitrella) *patens* phyllid development. *J. Bryol.* **41**, 314–321 (2019).

33. D. Poli, M. Jacobs, T. J. Cooke, Auxin regulation of axial growth in bryophyte sporophytes: Its potential significance for the evolution of early land plants. *Am. J. Bot.* **90**, 1405–1415 (2003).

34. T. A. Bennett, M. M. Liu, T. Aoyama, N. M. Bierfreund, M. Braun, Y. Coudert, R. J. Dennis, D. O'Connor, X. Y. Wang, C. D. White, E. L. Decker, R. Reski, C. J. Harrison, Plasma membrane-targeted PIN proteins drive shoot development in a moss. *Curr Biol.* **24**, 2776–2785 (2014).

35. T. Viaene, K. Landberg, M. Thelander, E. Medvecka, E. Pederson, E. Feraru, E. D. Cooper, M. Karimi, C. F. Delwiche, K. Ljung, M. Geisler, E. Sundberg, J. Friml, Directional auxin transport mechanisms in early diverging land plants. *Curr. Biol.* **24**, 2786–2791 (2014).

36. H. Kato, R. Nishihama, D. Weijers, T. Kohchi, Evolution of nuclear auxin signaling: Lessons from genetic studies with basal land plants. *J. Exp. Bot.* **69**, 291–301 (2018).

37. M. Thelander, K. Landberg, E. Sundberg, Auxin-mediated developmental control in the moss *Physcomitrella patens*. *J. Exp. Bot.* **69**, 277–290 (2018).

38. V. M. Lüth, C. Rempfer, N. van Gessel, O. Herzog, M. Hanser, M. Braun, E. L. Decker, R. Reski, A *Physcomitrella* PIN protein acts in spermatogenesis and sporophyte retention. *New Phytol.* **237**, 2118–2135 (2023).

39. S. Strauss, A. Runions, B. Lane, D. Eschweiler, N. Bajpai, N. Trozzi, A.-L. Routier-Kierzkowska, S. Yoshida, S. Rodrigues da Silveira, A. Vijayan, R. Tofanelli, M. Majda, E. Echevin, C. Le Gloanec, H. Bertrand-Rakusova, M. Adibi, K. Schneitz, G. W. Bassel, D. Kierzkowski, J. Stegmaier, M. Tsiantis, R. S. Smith, Using positional information to provide context for biological image analysis with MorphoGraphX 2.0. *eLife* **11**, e72601 (2022).

40. R. Di Mambro, M. De Ruvo, E. Pacifici, E. Salvi, R. Sozzani, P. N. Benfey, W. Busch, O. Novak, K. Ljung, L. Di Paola, A. F. M. Marée, P. Costantino, V. A. Grieneisen, S. Sabatini, Auxin minimum triggers the developmental switch from cell division to cell differentiation in the *Arabidopsis* root. *Proc. Natl. Acad. Sci. U.S.A.* **114**, E7641–E7649 (2017).

41. J. Cammarata, A. H. K. Roeder, M. J. Scanlon, The ratio of auxin to cytokinin controls leaf development and meristem initiation in *Physcomitrium patens*. *J. Exp. Bot.* **74**, 6541–6550 (2023).

42. K. Landberg, J. Šimura, K. Ljung, E. Sundberg, M. Thelander, Studies of moss reproductive development indicate that auxin biosynthesis in apical stem cells may constitute an ancestral function for focal growth control. *New Phytol.* **229**, 845–860 (2021).

43. Y. Zhao, Auxin biosynthesis: A simple two-step pathway converts tryptophan to indole-3-acetic acid in plants. *Mol. Plant* **5**, 334–338 (2012).

44. M. Thelander, K. Landberg, A. Muller, G. Cloarec, N. Cunniffe, S. Huguet, L. Soubigou-Taconnat, V. Brunaud, Y. Coudert, Apical dominance control by TAR-YUC-mediated auxin biosynthesis is a deep homology of land plants. *Curr. Biol.* **32**, 3838–3846.e5 (2022).

45. M. Thelander, K. Landberg, E. Sundberg, Minimal auxin sensing levels in vegetative moss stem cells revealed by a ratiometric reporter. *New Phytol.* **224**, 775–788 (2019).

46. M. Adamowski, J. Friml, PIN-dependent auxin transport: Action, regulation, and evolution. *Plant Cell* **27**, 20–32 (2015).

47. M. Sauer, J. Balla, C. Luschnig, J. Wiśniewska, V. Reinöhl, J. Friml, E. Benková, Canalization of auxin flow by Aux/IAA-ARF-dependent feedback regulation of PIN polarity. *Genes Dev.* **20**, 2902–2911 (2006).

48. D. J. Cove, "Regulation of Development in the Moss, *Physcomitrella patens*," in *Development: The Molecular Genetic Approach.*, V. E. A. Russo, S. Brody, D. Cove, S. Ottolenghi, Eds. (Springer Berlin Heidelberg, 1992), pp. 179–193; https://doi.org/10.1007/978-3-642-77043-2_13.

49. H. Tang, K.-J. Lu, Y. Z. Zhang, Y.-L. Cheng, S.-L. Tu, J. Friml, Divergence of trafficking and polarization mechanisms for PIN auxin transporters during land plant evolution. *Plant Commun.* **5**, 100669 (2024).

50. H. Tang, L.-H. Chen, J. Friml, Auxin fluctuation and PIN polarization in moss leaf cell reprogramming. *Plant Cell Physiol.* **66**, 658–667 (2025).

51. B. Mohanasundaram, A. J. Bhide, S. Palit, G. Chaturvedi, M. Lingwan, S. K. Masakapalli, A. K. Banerjee, The unique bryophyte-specific repeat-containing protein SHORT-LEAF regulates gametophore development in moss. *Plant Physiol.* **187**, 203–217 (2021).

52. J. Abitbol-Spangaro, G. Cloarec, A. Muller, S. Hallet, C. Boulogne, C. Gillet, V. Schmidt, P. I. Dobrev, R. Skokan, V. Couvreur, J. de Keijzer, C. Godin, Y. Coudert, Robust branch patterning in moss shoots via symplasmic auxin diffusion. *Curr. Biol.* **21**, 5238–5251 (2025).

53. Y. Coudert, W. Palubicki, K. Ljung, O. Novak, O. Leyser, C. J. Harrison, Three ancient hormonal cues co-ordinate shoot branching in a moss. *eLife* **4**, 06808 (2015).

54. X. Han, T. K. Hyun, M. Zhang, R. Kumar, E. Koh, B.-H. Kang, W. J. Lucas, J.-Y. Kim, Auxin-callose-mediated plasmodesmal gating is essential for tropic auxin gradient formation and signaling. *Dev. Cell* **28**, 132–146 (2014).

55. N. L. Mellor, U. Voß, G. Janes, M. J. Bennett, D. M. Wells, L. R. Band, Auxin fluxes through plasmodesmata modify root-tip auxin distribution. *Development* **147**, dev181669 (2020).

56. N. M. Linh, E. Scarpella, Leaf vein patterning is regulated by the aperture of plasmodesmata intercellular channels. *PLoS Biol.* **20**, e3001781 (2022).

57. G. Zotz, K. Wilhelm, A. Becker, Heteroblasty—A review. *Bot. Rev.* **77**, 109–151 (2011).

58. A. Gómez-Felipe, E. Branchini, B. Wang, M. Marconi, H. Bertrand-Rakusová, T. Stan, J. Burkiewicz, S. de Folter, A.-L. Routier-Kierzkowska, K. Wabnik, D. Kierzkowski, Two orthogonal differentiation gradients locally coordinate fruit morphogenesis. *Nat. Commun.* **15**, 2912 (2024).

59. L. Moubayidin, L. Ostergaard, Dynamic control of auxin distribution imposes a bilateral-to-radial symmetry switch during gynoecium development. *Curr. Biol.* **24**, 2743–2748 (2014).

60. E. Scarpella, D. Marcos, J. Friml, T. Berleth, Control of leaf vascular patterning by polar auxin transport. *Genes Dev.* **20**, 1015–1027 (2006).

61. G. J. Mitchison, A model for vein formation in higher plants. *Proc. R. Soc. Lond. Biol. Sci.* **207**, 79–109 (1980).

62. Y. Ge, Y. Gao, Y. Jiao, Y. Wang, A conserved module in the formation of moss midribs and seed plant axillary meristems. *Sci. Adv.* **8**, eadd7275 (2022).

63. M. Ishikawa, A. Fujiwara, K. Kosetsu, Y. Horiuchi, N. Kamamoto, N. Umakawa, Y. Tamada, L. Zhang, K. Matsushita, G. Palfalvi, T. Nishiyama, S. Kitasaki, Y. Masuda, Y. Shiroza, M. Kitagawa, T. Nakamura, H. Cui, Y. Hiwatashi, Y. Kabeya, S. Shigenobu, T. Aoyama, K. Kato, T. Murata, K. Fujimoto, P. N. Benfey, M. Hasebe, R. Kofuji, GRAS transcription factors regulate cell division planes in moss overriding the default rule. *Proc. Natl. Acad. Sci. U.S.A.* **120**, e2210632120 (2023).

64. R. Skokan, E. Medvecká, T. Viaene, S. Vosolsobě, M. Zwiewka, K. Müller, P. Skůpa, M. Karady, Y. Zhang, D. P. Janacek, U. Z. Hammes, K. Ljung, T. Nodzyński, J. Petrášek, J. Friml, PIN-driven auxin transport emerged early in streptophyte evolution. *Nat. Plants* **5**, 1114–1119 (2019).

65. A. Paterlini, Uncharted routes: Exploring the relevance of auxin movement via plasmodesmata. *Biol. Open* **9**, bio055541 (2020).

66. Y. Coudert, O. Novák, C. J. Harrison, A KNOX-cytokinin regulatory module predates the origin of indeterminate vascular plants. *Curr. Biol.* **29**, 2743–2750.e5 (2019).

67. W. Yang, C. Schuster, C. T. T. Beahan, V. Charoensawan, A. Peaucelle, A. Bacic, M. S. S. Doblin, R. Wightman, E. M. M. Meyerowitz, Regulation of meristem morphogenesis by cell wall synthases in arabidopsis. *Curr. Biol.* **26**, 1404–1415 (2016).

68. L. Willis, Y. Refahi, R. Wightman, B. Landrein, J. Teles, K. C. Huang, E. M. Meyerowitz, H. Jönsson, Cell size and growth regulation in the *Arabidopsis thaliana* apical stem cell niche. *Proc. Natl. Acad. Sci. U.S.A.* **113**, E8238–E8246 (2016).

69. D. R. Mallett, M. Chang, X. Cheng, M. Bezanilla, Efficient and modular CRISPR-Cas9 vector system for *Physcomitrella patens*. *Plant Direct* **3**, e00168 (2019).

70. J. Miao, D. Guo, J. Zhang, Q. Huang, G. Qin, X. Zhang, J. Wan, H. Gu, L.-J. Qu, Targeted mutagenesis in rice using CRISPR-Cas system. *Cell Res.* **23**, 1233–1236 (2013).

71. J.-P. Concordet, M. Haeussler, CRISPOR: Intuitive guide selection for CRISPR/Cas9 genome editing experiments and screens. *Nucleic Acids Res.* **46**, W242–W245 (2018).

72. N. Fernandez-Pozo, F. B. Haas, R. Meyberg, K. K. Ullrich, M. Hiss, P.-F. Perroud, S. Hanke, V. Kratz, A. F. Powell, E. F. Vesty, C. G. Daum, M. Zane, A. Lipzen, A. Sreedasyam, J. Grimwood, J. C. Coates, K. Barry, J. Schmutz, L. A. Mueller, S. A. Rensing, PEATmoss (*Physcomitrella* Expression Atlas Tool): A unified gene expression atlas for the model plant *Physcomitrella patens*. *Plant J.* **102**, 165–177 (2019).

73. P.-F. Perroud, F. B. Haas, M. Hiss, K. K. Ullrich, A. Alboresi, M. Amirebrahimi, K. Barry, R. Bassi, S. Bonhomme, H. Chen, J. C. Coates, T. Fujita, A. Guyon-Debast, D. Lang, J. Lin, A. Lipzen, F. Nogué, M. J. Oliver, I. P. de León, R. S. Quatrano, C. Rameau, B. Reiss, R. Reski, M. Ricca, Y. Saidi, N. Sun, P. Szövényi, A. Sreedasyam, J. Grimwood, G. Stacey, J. Schmutz, S. A. Rensing, The *Physcomitrella patens* gene atlas project: Large-scale RNA-seq based expression data. *Plant J.* **95**, 168–182 (2018).

74. P. Barbier de Reuille, A.-L. Routier-Kierzkowska, D. Kierzkowski, G. W. Bassel, T. Schüpbach, G. Tauriello, N. Bajpai, S. Strauss, A. Weber, A. Kiss, A. Burian, H. Hofhuis, A. Sapala, M. Lipowczan, M. B. Heimlicher, S. Robinson, E. M. Bayer, K. Basler, P. Koumoutsakos, A. H. K. Roeder, T. Aegerter-Wilmsen, N. Nakayama, M. Tsiantis, A. Hay, D. Kwiatkowska, I. Xenarios, C. Kuhlemeier, R. S. Smith, MorphoGraphX: A platform for quantifying morphogenesis in 4D. *eLife* **4**, 5864 (2015).

75. N. Wojciechowska, K. Marzec-Schmidt, E. M. Kalemba, A. Zarzyńska-Nowak, A. M. Jagodziński, A. Bagniewska-Zadworna, Autophagy counteracts instantaneous cell death during seasonal senescence of the fine roots and leaves in Populus trichocarpa. *BMC Plant Biol.* **18**, 260 (2018).

76. C. S. Galvan-Ampudia, G. Cerutti, J. Legrand, G. Brunoud, R. Martin-Arevalillo, R. Azais, V. Bayle, S. Moussu, C. Wenzl, Y. Jaillais, J. U. Lohmann, C. Godin, T. Vernoux, Temporal integration of auxin information for the regulation of patterning. *eLife* **9**, e55832 (2020).

77. G. Mosca, A. Eng, M. Adibi, S. Yoshida, B. Lane, L. Bergheim, G. Weber, R. S. Smith, A. Hay, Growth and tension in explosive fruit. *Curr. Biol.* **34**, 1010–1022.e4 (2024).

78. G. W. Bassel, P. Stamm, G. Mosca, P. Barbier de Reuille, D. J. Gibbs, R. Winter, A. Janka, M. J. Holdsworth, R. S. Smith, Mechanical constraints imposed by 3D cellular geometry and arrangement modulate growth patterns in the *Arabidopsis* embryo. *Proc. Natl. Acad. Sci. U.S.A.* **111**, 8685–8690 (2014).
